# Genomic structure of the horse major histocompatibility complex class II region resolved using PacBio long-read sequencing technology

**DOI:** 10.1038/srep45518

**Published:** 2017-03-31

**Authors:** Agnese Viļuma, Sofia Mikko, Daniela Hahn, Loren Skow, Göran Andersson, Tomas F. Bergström

**Affiliations:** 1 Department of Animal Breeding and Genetics, Swedish University of Agricultural Sciences (SLU), Box 7023, 750 07 Uppsala, Sweden; 2 Department of Veterinary Integrative Biosciences, College of Veterinary Medicine, Texas A&M University, College Station, TX 77843, USA.

## Abstract

The mammalian Major Histocompatibility Complex (MHC) region contains several gene families characterized by highly polymorphic loci with extensive nucleotide diversity, copy number variation of paralogous genes, and long repetitive sequences. This structural complexity has made it difficult to construct a reliable reference sequence of the horse MHC region. In this study, we used long-read single molecule, real-time (SMRT) sequencing technology from Pacific Biosciences (PacBio) to sequence eight Bacterial Artificial Chromosome (BAC) clones spanning the horse MHC class II region. The final assembly resulted in a 1,165,328 bp continuous gap free sequence with 35 manually curated genomic loci of which 23 were considered to be functional and 12 to be pseudogenes. In comparison to the MHC class II region in other mammals, the corresponding region in horse shows extraordinary copy number variation and different relative location and directionality of the *Eqca-DRB, -DQA, -DQB* and *–DOB* loci. This is the first long-read sequence assembly of the horse MHC class II region with rigorous manual gene annotation, and it will serve as an important resource for association studies of immune-mediated equine diseases and for evolutionary analysis of genetic diversity in this region.

More than twenty years ago, John Trowsdale described the Major Histocompatibility Complex (MHC) as “the center of the immune universe” to emphasize its importance for initiating specific immune responses^1^. Indeed, the statement has been validated by the extremely large number of immune-mediated diseases associated to the human MHC (for review see ref. 2). The MHC is gene dense and characterized by considerable genomic complexity. Genes in the complex are traditionally divided into the MHC class I, -II and –III regions. The classical MHC class II molecules (DR, DQ and DP) are heterodimeric cell surface glycoproteins consisting of α- and β-chains that bind and present exogenous peptides to circulating CD4^+^ T-helper cells, thereby initiating immune responses against pathogens^3^.

The human MHC, denoted Human Leucocyte Antigen (HLA), spans approximately 3.6 Mbp on the short arm of chromosome six (6p21)^4^. Association of HLA genetic variation with increased susceptibility or resistance to inflammatory and autoimmune diseases, such as type 1 diabetes^5^,^6^, narcolepsy^7^, coeliac disease^6^ and rheumatoid arthritis^8^ is well documented. Furthermore, in the co-evolution of host-pathogen interactions, MHC variation has also been shown to influence resistance or susceptibility to infectious diseases. A classical example of this is the association of *HLA-B53* with resistance to severe malaria in West Africa^9^,^10^.

In the horse, MHC association to immune-mediated diseases (e.g. Insect Bite Hypersensitivity^11^,^12^,^13^, Equine Sarcoids^14^ and Equine Recurrent Uveitis^15^), has also been demonstrated. The horse MHC region, designated ELA, is located on chromosome 20q14-q22^16^ and, as expected, contains highly polymorphic genes encoding classical MHC class II molecules^17^,^18^,^19^,^20^,^21^. The horse genome reference sequence (EquCab2) from the Thoroughbred mare Twilight was released in 2007^22^. To complement the assembly of EquCab2, Children’s Hospital Oakland Research Institute created a BAC clone library CHORI-241^23^ from the Thoroughbred male Bravo. Using hybridization screening of CHORI-241 library, BAC clones constituting a minimum tiling-path of the horse MHC was identified and an ordered BAC contig map was constructed^24^,^25^.

Generating a reliable high-quality *de novo* sequence assembly using traditional Sanger-sequencing or short read next-generation sequencing technologies over a complex region, such as the horse MHC class II, is inherently difficult due to the many multigene families with copy number variation of paralogous loci, long repetitive elements and extensive allelic polymorphism.

Recent studies have shown that the long-read single molecule, real-time (SMRT) sequencing technology from Pacific Bioscience (PacBio) can be successfully applied to resolve complex genomic regions and improve genome assemblies^26^,^27^,^28^,^29^. The purpose of this study was to provide an enhanced and carefully annotated reference sequence of the MHC class II region.

## Results

### Long-read sequencing and assembly of the horse MHC class II region

To resolve the repetitive complexity and genomic structure of the horse MHC class II region (~1.2 Mbp), we sequenced eight large-insert clones of the CHORI-241 Horse BAC library with PacBio long-read single molecule, real-time (SMRT) sequencing technology. These eight clones, derived from the male horse Bravo, had previously been shown to constitute a minimum tiling-path over the horse MHC class II region^24^,^25^. The strategy of sequencing single BAC clones resulted in mean read coverage exceeding 300-fold for each BAC clone, while the pooled sequencing strategy resulted in a mean coverage ranging from 108- to 287-fold (Table 1). The depth of each BAC assembly is shown in detail in Supplementary Fig. S1.

**Figure 1:**
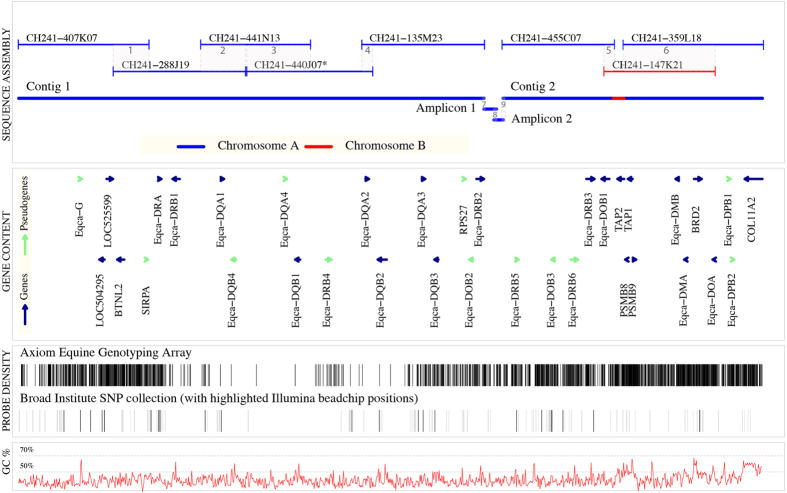
Physical map of the horse MHC class II region. Sequence assembly of eight BAC clones constituting a minimum-tiling path. Consecutively numbered junctions are indicated with grey shading and Arabic numerals. The BAC clone assembly forms two contigs, Contig 1 and 2, joined by Amplicons 1 and 2. Blue and red colours indicates two the homologous chromosomes 20 of the horse Bravo, Chromosome A and Chromosome B. An asterisk indicates the partially sequenced BAC clone. Gene content illustrates the location and orientation of all detected genes (blue) and pseudogenes (green) with transcriptional directionality indicated by arrows. Probe density describes the variation in the probe coverage over the MHC class II region. Probe mapping locations of the Axiom Equine Genotyping array are displayed as black lines. Broad Institute SNP collection probe locations are displayed as grey lines and Illumina beadchip positions are highlighted in black. GC% describes the percentage of G and C nucleotides in 1 kbp windows.

To produce a *de novo* assembly of the horse MHC class II region, we identified overlapping regions (junctions) of the eight sequenced BAC clones. The six detected junctions ranged from 13,475 to 141,592 bp (Table 2, junctions 1 to 6). Based on the previously published minimum tiling-path of the horse MHC class II region, we expected the final clone assembly to be a single contig. However, contrary to expectations, the BAC clones CH241-135M23 and CH241-455C07 were found to be non-overlapping. Thus, the assembly of the eight BAC clones resulted in two contigs, designated Contig 1 and Contig 2 (Fig. 1). We assembled Contig 1 from five (CH241-407K07, -288J19, -441N13, -440J07 and -135M23) and Contig 2 from three (CH241-455C07, -359L18 and -147K21) BAC clones.

We observed more than a 50-fold higher proportion of mismatched bases in junctions 5 (clone CH241-147K21 and CH241-455C07) and 6 (CH241-147K21 and CH241-359L18) as compared to junctions 1 to 4 (Table 2). To investigate if the higher proportion of mismatched bases could be explained by heterozygosity in the genomic sequence of the horse Bravo, five regions with dense SNP variation (in total 46 SNPs) and four small INDELs (1–2 bp) in junctions 5 and 6 were Sanger-sequenced. The results showed that 45 of the 46 SNPs and three of the four small INDELs were heterozygous in genomic DNA of the horse Bravo (detailed results in Supplementary Table S1). Agarose gel electrophoresis of a PCR product also confirmed that Bravo is heterozygous for the 247 bp INDEL. The sequence of this INDEL was identified as an Equine Repetitive Element 1 (ERE1). Insertion polymorphism of this transposable element has been previously reported^30^ and is considered to represent a recent integration in the horse genome.

The increased proportion of mismatched bases in junctions 5 and 6, and Sanger-sequencing results suggested that the BAC clones CH241-455C07 and CH241-359L18 originated from the same chromosome 20 of the horse Bravo (indicated as Chromosome A in Fig. 1) and that the BAC clone CH241-147K21 was derived from the other homologous chromosome 20 (indicated as Chromosome B in Fig. 1). Assuming that all observed SNPs in junctions 5 and 6 are reflecting “true” nucleotide difference, the genetic distance of the two chromosomes was estimated to be 0.0023 (p-distance). Applying a mutation rate of 7.242 × 10^−9^ per site per generation^31^, we estimated the divergence time between the two chromosomes to be approximately one million years, which is comparable to the divergence time of alleles from the same allelic lineage in human^32^. The entire list of heterozygous positions is provided in the Supplementary Dataset 1. Junctions 1 to 4 showed a low proportion of mismatched bases (≤0.004%), suggesting that all BAC clones in Contig 1 originated from the same chromosome (i.e. Chromosome A in Fig. 1).

The gap separating Contig 1 and 2 was closed using PacBio long-read SMRT sequencing of two overlapping long-range PCR amplicons (detailed information in Supplementary Table S2 and Fig. S2). The amplicon sequencing resulted in five error corrected full-length reads (19,701 bp) for Amplicon 1 and 47 error corrected full-length reads (14,649 bp) for Amplicon 2. The final assembly of Contig 1 and 2 together with Amplicon 1 and 2 yielded three extra junctions (Table 2, Junctions 7 to 9).

A single 1 bp INDEL difference and no base pair substitutions were observed in the junction 8 and no mismatched base pairs were observed in junctions 7 and 9. These junctions connected the Contig 1 with Contig 2 into a continuous gap free 1,165,328 bp genomic sequence, spanning the entire classical MHC class II region and part of a ‘gene desert’ located upstream of the class II region (Fig. 1). With the exception of a region between the BAC clones CH241-455C07 and -359L18 (denoted Chromosome B in Fig. 1) that partially spans the *TAP2* locus, the resulting genomic sequence appears to be derived from a single chromosome.

### Gene Annotation of the horse MHC class II assembly

**Figure 2:**
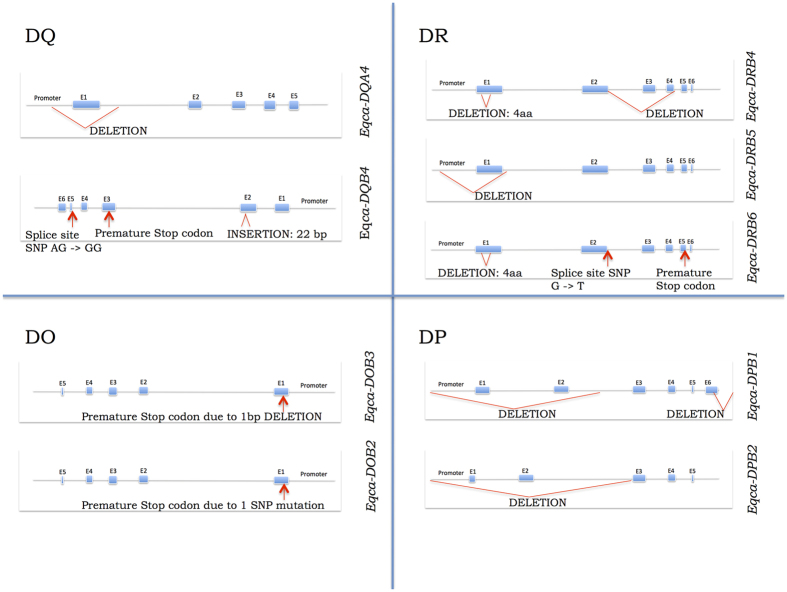
MHC class II pseudogenes. The schematic structure of the horse MHC class II pseudogenes with the disrupting mutations indicated in red.

In total, we identified 35 genes in the final assembly of the horse MHC class II region (Fig. 1). The manually curated gene models are described in a gff2 file format (Supplementary Dataset 2). Of the 35 identified loci, 20 had undisrupted open reading frames supported by mRNA data (*Eqca-DRA1, -DRB1, -DQA1, -DQB1, -DQA2, -DQB2, -DQA3, -DQB3, -DRB2, -DRB3, -DOB1, TAP2, PSMB8, TAP1, PSMB9, Eqca-DMA, -DMB, BRD2, Eqca-DOA* and *COL11A2*) while three loci had undisrupted open reading frames without supporting mRNA evidence (*LOC504295, LOC525599, BTNL2*). We classified ten loci as unprocessed pseudogenes as the predicted exon structure displayed disruptive mutations in the open reading frame (*SIRPA, Eqca-DQB4, -DQA4, -DRB4, -DOB2, -DRB5, -DOB3, -DRB6, -DPB1, -DPB2*) and one locus was identified as a processed ribosomal protein pseudogene *RPS27*. All pseudogenes except *SIRPA* and *RPS27* were members of multigene families (Fig. 2). In addition, we identified a single pseudogene with 80% sequence similarity to the human non-classical *HLA-G* class I locus. Gene content of each BAC clone is described in Supplementary Table S3.

### Classical MHC class II genes

Six *Eqca*-*DRB* loci of which three are functional, were identified in the equine class II region. A single *Eqca-DRA1* locus was located on the forward strand in a tail-to-tail orientation with *Eqca-DRB1*, separated by approximately 17.5 kbp. The other *Eqca-DRB* genes were located on the forward strand. We observed that intron 1 of *Eqca-DRB3* (derived from the BAC-clone CH241-455C07) had a potentially disrupting SNP in the splice acceptor site (AG > TG). All other sequenced *DRB* genes and the equivalent positions in EquCab2 had the functional splice acceptor site (AG).

We identified four pairs of *Eqca-DQA* and *-DQB* genes. Each pair was organised in tail-to-tail orientation (Fig. 1). Alignment of the predicted protein sequences of all functional *Eqca-DQB* genes to *HLA-DQB1 and –DQB2* showed that exon 6 of the *Eqca-DQB3* encoded a protein three amino acids longer than that encoded by the homologous HLA genes (Supplementary Fig. S3). Further, a nucleotide sequence comparison of the *Eqca-DQB1* and *-DQB3* locus revealed a ~400 bp deletion spanning the protein coding sequence of the conserved exon 6 and part of intron 5 of the *Eqca-DQB3* gene. However, the spliced alignment of a single mRNA read^33^ at this locus suggested that a downstream 3′UTR sequence might be used as an alternative exon 6 (see Supplementary Fig. S4).

The coding sequences of the functional DR and DQ genes were compared to publicly available MHC alleles at MHC-IPD^34^ and the detailed results and alignments can be found in the Supplementary Dataset 3 and Table S4.

### Non-classical MHC class II genes

The annotation resulted in the identification of four functional non-classical MHC class II genes (*Eqca-DOA, -DOB, -DMA* and *-DMB)* encoding the DO and DM molecules. When comparing the CDS of our gene model for *Eqca-DOA* to the RefSeq^35^ annotation of *HLA-DOA*, we observed a length difference of 36 bp (see Supplementary Dataset 3). The alignment of the CDS of *Eqca-DOA* to *HLA-DOA* and the corresponding region in EquCab2 revealed a SNP (GT > GC) at the splice site at the 5′-end of intron 5 (Supplementary Fig. S5). By aligning mRNA evidence at *Eqca-DOA* locus, we located an alternative canonical GT splice site, 36 bp downstream the human splice site, which predicts a 36 bp larger exon 4 in *Eqca-DOA*.

### Density and distribution of horse SNP locations

To facilitate the experimental design and result interpretation from genome wide association studies (GWAS) we investigated the density of the probe sequences of Axiom Equine Genotyping Array and a horse SNP collection from the Broad Institute spanning the Bravo MHC class II region (Fig. 1). Overall, the results showed that the horse MHC class II region was densely covered with SNPs (Fig. 1). However, several classical class II genes, such as *Eqca-DQA1, -DQB1, -DQA2* and *-DQB2* were covered poorly.

### Sequence comparison to EquCab2 assembly over the MHC class II region

We aligned each PacBio sequenced BAC clone insert to the MHC class II region of EquCab2. A summary of sequence differences observed from the alignments is described in Table 3. The estimated genetic distance (p-distance) and the two sequence identity scores, per-base and per-event identity, showed that clone CH241-455C07 was the least similar and clone CH241-288J19 was the most similar to EquCab2. It is important to note that we were not able to compare sequences that coincide with the gap regions of EquCab2. These regions were excluded from the analyses.

**Figure 3:**
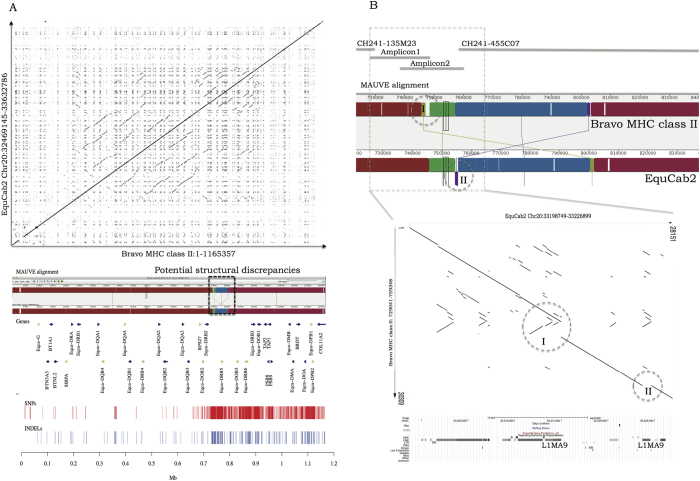
The sequence comparison of the long-read sequenced Bravo MHC class II and Twilight reference assembly EquCab2. Genomic coordinates used in the comparisons are displayed on the corresponding axes of the dot-plot and the MAUVE alignment. The homologous blocks in the MAUVE alignment are displayed in similar colours and connected with a line and no homology regions are showed as white areas. (**A**) A dot-plot and MAUVE alignment illustrating the overall comparison of the two genomic sequences. The region with potential structural discrepancies is highlighted with a black dashed square on the MAUVE alignment. The SNP and INDEL density is displayed below the alignment together with the gene content. (**B**) Detailed illustration of the region harbouring the potential structural discrepancies. Locations of the two structural discrepancies are indicated with dashed circles in the MAUVE alignment and in the dot-plot. The assembly details are displayed on the top of the MAUVE alignment and UCSC genome browser repetitive element annotation for EquCab2 is displayed below.

To further investigate structural differences between our final assembly (Bravo MHC Class II) and EquCab2, we analysed dot-plots and created a MAUVE alignment. The dot-plot analysis of the entire horse MHC class II region did not reveal any major structural discrepancies (Fig. 3A). However, the MAUVE alignment pinpointed a region with potential structural discrepancies between these two sequences. The overview of SNP and INDEL density of the entire region revealed a considerable increase in density starting with the region of the third intron of the *Eqca-DRB2* gene (Fig. 3A). Detailed analysis of the region harbouring the potential structural discrepancies (Fig. 3B) indicated two ~900 bp regions, designated as I and II with different relative location. The genomic sequence of these regions corresponded to a non-LTR retrotransposon *L1MA9* that belong to L1 LINE family.

### MHC class II structure in mammals

There is a limited number of placental mammals with reliable MHC class II assembly and annotation that can be used to investigate structural differences. Considering this, we chose to compare the MHC class II region of seven mammals from the clade Boreoeutheria that contains the two taxa Euarchontoglires (represented by mouse and human) and Laurasiatheria (represented by horse, pig, cattle, domestic dog and cat). The divergence estimates of these species^36^, chromosomal location and directionality of the MHC class II region can be found in Supplementary Fig. S6.

**Figure 4:**
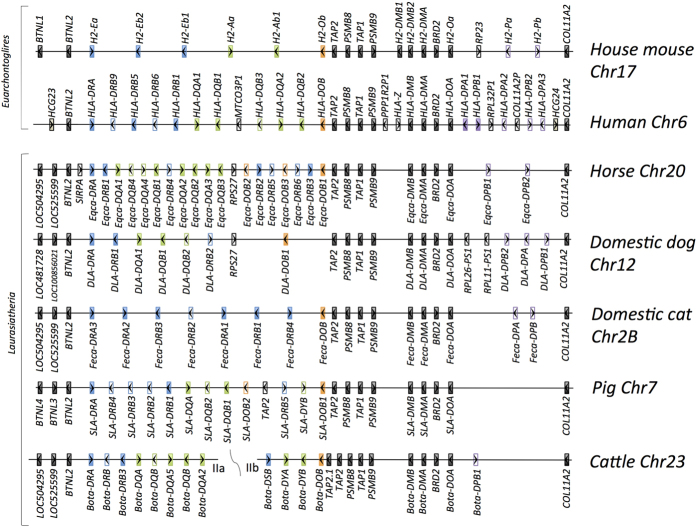
Comparison of MHC class II structure in mammals. The schematic (not to scale) gene order comparison of the horse MHC class II region to house mouse and human, illustrated above the horse, and to cattle, pig, domestic cat and dog, illustrated below the horse. Genes coding for DR, DQ, DOB and DP molecules are coloured in blue, green, orange and purple, respectively. The highly conserved genes are coloured in black. Gene *Eqca-G* was not included in this comparison, because it does not belong to MHC class II region. For better overview, the cattle MHC class IIb sequence was inverted. The nomenclature of the classical MHC class II genes was adjusted based on the MHC Nomenclature report^70^ and the allele nomenclature in MHC-IPD database. Note that the nomenclature does not reflect orthology.

We observed a high degree of conservation among the seven species in three distinct locations across the MHC class II region – the region from *TAP2* to *Eqca-DOA*, as well as the two genes *BTNL2* and *COL11A2* (Fig. 4). In the five laurasiatherian mammals the conserved region of *BTNL2* gene also included the genes *LOC504295* and *LOC525599*.

The number of paralogous DR and DQ genes was found to be variable among the compared species. For example, horse has four *Eqca-DQA* and *–DQB* loci, while there is a complete absence of DQ genes in cat. In horse, the six identified *Eqca-DRB* loci were distributed across a region spanning ~0.7 Mbp with intervening genes separating the *Eqca-DRB* loci. All *DRB* genes in human, mouse, domestic cat, pig and cattle were clustered together and located on the reverse strand in tail-to-tail orientation to the *DRA* gene. The phylogenetic analysis of mammalian DQ and DR gene families indicated a species-specific clustering of most of the paralogous loci (Supplementary Fig. S7). In contrast to HLA, we found no evidence for the existence of functional *Eqca-DPA* or -*DPB* genes in horse or in the other compared species. All compared species, aside from pig, showed variable number of DP pseudogenes located between the highly conserved *Eqca-DOA* and *COL11A2* loci.

Interestingly, the processed pseudogene *RPS27* upstream the *Eqca-DOB* gene was present in domestic dog, but appears to be absent in all other investigated species. Domestic dog showed additional similarities to horse, such as the existence of only a single functional *DRB* gene located on the reverse strand in a cluster with the *DRA* gene and an inverted *DRB* pseudogene (*Eqca-DRB4* and *DLA-DRB2*) located downstream of a DQ cluster. All of the compared laurasiatherian species had a single inverted *DRB* gene (*DLA-DRB2, Feca-DRB4, SLA-DRB5* and *Bota-DSB* gene) and horse showed five inverted *Eqca-DRB* genes. However, no *DRB* inversions were observed in human and mouse.

## Discussion

To resolve the complex genomic structure of the horse MHC class II region, we sequenced eight BAC clones from the CHORI-241 library using long-read single molecule, real-time (SMRT) sequencing technology from Pacific Bioscience (PacBio)^37^. For each BAC we produced more than a 100-fold average sequencing coverage, ranging from 108- (CH241-440J07) to 572-fold (CH241-455C07). The PacBio generated errors are of random nature^38^, and by exceeding a threshold of 80-fold read coverage a perfect consensus sequence can be produced^39^. Thus, we expected the obtained coverage to be sufficient for the assembly of high quality consensus sequence with low error rate.

We have concluded that in contrast to all other sequenced clones, clone CH241-147K21 represents the genomic sequence from the alternative homologous chromosome 20 (Chromosome B in Fig. 1) with genetic distance of 0.23% (SNP-based distance). Therefore, we hypothesise that on average a SNP should be observed every 440 bp, if two BAC clone insert sequences are derived from different chromosomes. Due to the lack of SNP differences and an extremely low percentage mismatch of small INDELs in the junctions of these clones, we suggest that clones CH241-407K07, -288J19, -441N13, 440J07 and -135M23 are derived from the same chromosome. We also propose that the small INDELs observed in these junctions represent the PacBio INDEL error rate that have persisted the deep coverage and it is estimated to be very low (0.003%).

We were able to bridge the gap of the non-overlapping BAC clones (CH241-135M23 and CH241-455C07) with two long-range PCR amplicons that were full-length sequenced using SMRT sequencing technology. This confirmed the consecutive order of two non-overlapping clones. As we did not observe any base pair substitutions and only one single bp INDEL across the junctions 7 to 9 (4,557 bp in total), we suggest that Contig 1 is possibly derived from the same homologous chromosome 20 as clones CH241-455C07 and -359L18 (Chromosome A in Fig. 1).

The publicly available annotation of the horse MHC class II region in EquCab2 is based on automated gene prediction pipelines, cross-species alignment and the full-length mRNA sequences. As a consequence of the limited availability of equine mRNA sequence data and manual gene annotation, a fraction of previously predicted gene models are inaccurate and lack proper naming. Recently, mRNA sequencing data from several horse breeds and tissues were published^33^,^40^. These sequences were used to carefully validate predicted gene models, and, when possible, to extend the 5′ and 3′ UTRs. The annotation resulted in the identification of 35 manually curated genomic loci of which 23 were considered to be functional and 12 to be pseudogenes.

In placental mammals, three types of classical MHC class II antigen presenting molecules (MHC-DR, -DQ and -DP) are known. In most mammals, the DR α chain is encoded by a single *Mhc-DRA* locus with limited polymorphism. In contrast, the β-chain of the DR molecules is encoded by one of several polymorphic *Mhc-DRB* loci. Similarly, the DQ and DP molecules are encoded by α- and β-chains from their respective *A* and *B* loci. In horse, only two types of antigen presenting molecules, DR and DQ, are known.

Three functional *Eqca-DRB* genes have previously been identified^41^ and the IPD-MHC database^34^ contains 13 alleles derived from these loci. However, the first intron of the Bravo *Eqca-DRB3* locus contained a disruptive acceptor splice site mutation in contrast to the corresponding acceptor splice site of the reference genome. The results suggest that the *Eqca-DRB3* locus harbours both functional protein coding alleles and at least one null allele. This is similar to the *HLA-DRB4* locus where null alleles have been identified^42^.

Evidence for at least two *Eqca-DQB*^43^ and two *-DQA*^18^ loci in horse was described more than a decade ago. Currently, the MHC-IPD database includes alleles from three *Eqca-DQA* and three -*DQB* loci. The results of our annotation are in concordance with the number of loci found in the MHC-IPD database, implying three functional *Eqca-DQA* and *-DQB* genes. However, all *Eqca-DQB3* alleles currently present in the MHC-IPD are derived from partially sequenced cDNAs (the 2^nd^, 3^rd^ and 4^th^ exon). We observed that the conserved protein coding sequence of exon 6 of the *Eqca-DQB3* gene has been deleted. In humans, a functional β-chain is encoded by six exons, where exon 1 encodes the signal peptide, exons 2 and 3 encode the 1^st^ and 2^nd^ domain, exon 4 encodes the membrane-spanning portion, and exon 5 and 6 encode the cytoplasmic tail of the β-chain^44^. Despite the deletion of exon 6, mRNA sequences of the *Eqca-DQB3* gene are reported in MHC-IPD database, suggesting that this gene is transcribed in the horse. In addition, a single spliced mRNA read provided evidence for the transcription of the conserved exons 2 to 5 and an alternative exon 6. More analysis is needed to determine whether the predicted alternative exon 6 is being translated into a functional protein.

Based on sequence comparison of each of the eight BAC clones to the EquCab2 assembly, we observed considerable variation in estimated genetic distance (Table 3). The observed variation may be caused by sequencing and assembly errors in either the PacBio assembly or the existing EquCab2 reference genome. However, the true genetic variation that exists between the two individuals used to produce these sequences *i.e*. the horse Bravo (BAC clone library donor) and Twilight (EquCab2 assembly donor) is unknown. This, together with the fact that the EquCab2 assembly was generated by a combination of whole genome shotgun sequencing of Twilight and end sequencing of more than 300 000 BAC clones from Bravo^22^, makes it difficult to draw reliable conclusions from the sequence comparison. Nevertheless, the results clearly highlight the increased divergence of the clone CH241-455C07, that is the clone with the deepest PacBio read coverage (572-fold). This clone covers a challenging region of MHC class II that harbours three *Eqca-DRB* and two *-DOB* copies, thereby complicating assembly of this region. Considering that both sequenced horses are half siblings derived from an inbred MHC homozygous horse herd^45^ of a horse breed well known for having high inbreeding coefficient and low breed diversity^46^ we would not expect to observe high within-breed variation. The PacBio full-length sequenced reads make us confident in the sequence assembly of the two long-range PCR amplicons that show sequence discrepancies involving LINE element *L1MA9*. Hence we imply that even though rare INDEL errors are present in Bravo MHC class II assembly, the majority of the observed discrepancies between Bravo MHC class II sequence and the reference genome assembly represent combination of both, *bona fide* sequence variation between these two horses and the assembly as well as sequencing errors of EquCab2.

A comparative genomics analysis of the MHC class II regions from seven different mammalian species was performed. Despite the long divergence time of the compared species (85-100 million years ago)^36^, we could observe conserved gene order among seven distantly related mammals for the genes *BTNL2, Eqca-DRA, TAP2, PSMB8, TAP1, PSMB9, HLA-DMB, HLA-DMA, BRD2, HLA-DOA* and *COL11A2*, suggesting an orthologous relationship among these genes in mammals (Fig. 4). As expected, for the classical MHC class II genes poorly conserved gene order was found, where each of the species showed a different number, order and directionality of genes and pseudogenes. In human, there are five main *HLA-DR* haplotypes (*DR1, DR51, DR52, DR8* and *DR53*) with variable number of *DRB* loci^47^ located in a single cluster in tail-to-tail orientation to a single *HLA-DRA* gene^48^. It remains to be seen if the plasticity of the human DR region with several DR haplotypes, is also present in the MHC of the horse. The other compared species in this study diverged from horse at least 85 million years ago. Considering the plasticity of the MHC class II genes^49^ and the accelerated birth-and-death evolution of multigene families^50^, it is likely that most of the gene duplications observed in horse occurred within the equid lineage. This was also supported by the topology of the phylogenetic trees of mammalian DR and DQ gene families, where all loci in horse were found in single clusters (Supplementary Fig. S7).

We would like to highlight an observation that all the compared laurasitherian mammals had a single inverted *DRB* gene, while this was not observed in human and mouse. This may potentially indicate that the inversion event occurred 85–100 million years ago, after the separation of Euarchontoglires but prior to the divergence of laurasitherian species. In horse, this ancestral inversion has evolved into a separate inverted *DRB* gene cluster, representing both functional genes and pseudogenes.

We have provided the first in-depth long-read sequenced and manually annotated MHC class II sequence of the order Perissodactyla. Long-read sequencing enabled us to resolve all difficult gap regions present in the current EquCab2 reference sequence and to identify the regions with possible errors that warrant further investigation. Manual gene annotation resulted in the discovery of several new loci and, together with comparative analysis, confirmed that locations of the MHC class II classical genes are distinctly different from the MHC class II region of other mammals. A continuous, well-annotated high quality “reference” sequence is essential for better experimental design of equine immune-mediated association studies, such as Insect Bite Hypersensitivity (Summer Eczema), Equine Sarcoids and Retinal Uveitis. The presented results will also be valuable for further evolutionary and genetic studies of the MHC class II region.

## Materials and Methods

### BAC clone culturing and DNA extraction

The eight Bacterial Artificial Chromosome (BAC) clones derived from the CHORI-241 Horse BAC library were propagated from LB agar stabs in LB-Agar high salt media with 25 μg/mL chloramphenicol. BAC-DNA was extracted with QIAGEN Large-Construct Kit (Qiagen, Hilden, Germany) according to the manufacturers’ protocol for clones CH241-147K21, -455C07, -135M23, -288J19 and -441N13. ATP-dependent nuclease treatment was omitted for clones CH241-440J07, -359L18 and -407K07 to obtain sufficient DNA yield for generating barcoded SMRTbell libraries.

### BAC clone sequencing and assembly

SMRTbell libraries were prepared at the Uppsala Genome Center (Science for Life Laboratory, Uppsala University) according to the Pacific Biosciences 1.0 template preparation kit manufacturer’s instructions (Pacific Biosciences 10 kb template preparation protocol). The circular BAC clone DNA (2000 ng) was fragmented randomly using the Hydroshear at speed code 13 for 20 cycles. Sheared DNA was end-repaired and adaptor-ligated. The sequencing libraries were quantified on Qubit fluorometer (Thermo Fisher Scientific) and library size was confirmed using the Bioanalyzer 12000 kit (Agilent Technologies). For BAC clones CH241-147K21, -455C07, -135M23 and -288J19, each library was loaded on a separate SMRT cells and sequenced on a PacBio RSII system with P4/C2 chemistry (single clone sequencing strategy). For BAC clones CH241-440J07, -359L18, -407K07 and -441N13 barcoded adapters were added (pooled sequencing strategy) and equimolar amounts of three clones per SMRT cell were pooled before sequencing on the PacBio RSII system with P6/C4 chemistry. The movie time for sequencing was 240 minutes for all clones. *De novo* assembly of the generated sequencing reads was made by the hierarchical genome-assembly process (HGAP) algorithm^51^.

To analyse coverage of the assembly, we aligned all filtered subreads to the final assembly of each sequenced BAC clone construct with bwa v0.7.13 mem algorithm^52^ followed by SAMtools v1.3 ‘depth’ and ‘stats’^53^ analysis. Next, we trimmed the ends of all assemblies of clones CH241-147K21, -455C07, -135M23, -288J19 and -441N13, with less than 100-fold coverage, of clones CH241-407K07 and -359L18 less than 30- fold coverage and no trimming for clone CH241-440J07. GC content was analysed with BEDtools v2.25.0^54^.

For each linear BAC construct assembly we identified the *EcoR*I cloning sites and removed the vector sequence. The two remaining segments of each linear BAC clone construct assembly were then joined using software GAP5 v1.2.14-r^55^ to generate a continuous insert sequence. The eight obtained insert sequences were further analysed with software GAP5 to detect overlapping regions (junctions) and the proportion of non-matching (mismatched) base pairs in these junctions.

### Long-range PCR amplicon sequencing

Two long-range PCR primer sets were designed to amplify the missing genomic region between clone CH241-135M23 and CH241-455C07. PCR primer sequences were designed with Primer3 v.0.4.0^56^ and can be found in Supplementary Table S5. As the template for amplification, 200 ng of genomic DNA from the horse Bravo was used for amplification with Roche Expand Long Template PCR System 3 (Roche Diagnostics, Basel, Switzerland). The final PCR reaction concentrations were: dNTP mix 450 μM, forward and reverse primer 300 nM (Amplicon 1) or 180 nM (Amplicon 2) each, PCR buffer with MgCl_2_ 2.75 mM and 0.81 μl of Expand Long Template Enzyme mix. Thermal cycling was performed on a GeneAmp™ PCR System 9700 (Thermo Fisher Scientific) according to manufacturer’s protocol with 60 °C annealing temperature for Amplicon 1 and 61 °C for Amplicon 2. Both PCR products were pooled in a single sample (1660 ng) and sequenced on a PacBio RSII system (Science for Life Laboratory, Uppsala University) with P6/C4 chemistry. Filtered error corrected reads were assigned to Amplicon 1 or Amplicon 2 based on forward and reverse primer sequences and the full length amplicon sequences were further error corrected with Canu v.1.0.^57^ using the excess of shorter sequencing reads. Amplicons were used to join clone CH241-135M23 and CH241-455C07 in a single continuous sequence using GAP5 software.

### Sequence annotation

To achieve a higher sensitivity in gene model discovery, we masked repetitive sequences present in the insert sequence of each BAC using the online version of RepeatMasker^58^. Masked sequences were subjected to automated and manual annotation. We analysed each assembled BAC clone insert sequence using the gene prediction software AUGUSTUS^59^ with the option of incorporating *extrinsic* data sources such as cDNAs, ESTs, RNA-seq data and homologous protein sequences. The detailed scheme, software and data set references used in the annotation pipeline are described in the Supplementary Fig. S8. Manual gene annotation was done following the Havana annotation guidelines^60^ and *Drosophila melanogaster* annotation review^61^. In the process of manual gene model annotation we evaluated the reliability and mapping quality of the mRNA evidence, splice site discrepancies, separated merged gene models of paralogous genes, start and stop codon positions, extended 5′ UTR regions and located correct 3′ end coordinates according to mRNA evidence. The final predicted open reading frames were translated with ExPASy (Swiss Institute of Bioinformatics, http://web.expasy.org/translate/) and used to search the human protein database using BLAST. Individual validated gene models were lifted over to the final non-repeat masked Bravo MHC class II assembly with a custom Perl script. Coding exons, 5′ and 3′ UTRs coinciding with the repetitive regions were extended based on to the mapping mRNA evidence.

Coding sequences of MHC class II genes and alleles at MHC-IPD database were aligned using EMBOSS Needle nucleotide alignment tool (EMBL-EBI, http://www.ebi.ac.uk/Tools/psa/emboss_needle/nucleotide.html).

Probe sequences from 670,796 markers of the Axiom Equine Genotyping Array (Affymetrix, Thermo Fisher Scientific) and the Broad Institute SNP collection (available at https://www.broadinstitute.org/ftp/distribution/horse_snp_release/v2/) were aligned to the final assembly with bwa -mem aligner v.0.7.13. Short 71 bp Axiom Equine Genotyping Array probe sequences were subjected to the stringent quality filtering, by removing overlapping probes and probes showing variation other than the tested SNP and sequences with mapping quality lower than 60.

### Comparison to the EquCab2 assembly

We extracted the genomic region of EquCab2 chr20:32469145-33632786 for comparative analysis. The region was identified by aligning the two ends of the Bravo MHC class II assembly to the EquCab2 assembly. We aligned each BAC clone insert sequence to the extracted EquCab2 sequence and generated match and mismatch statistics (base pair substitutions, deletions and insertions) using BLASR^62^ software. We removed mismatching positions in the alignment caused by the unknown nucleotides (gaps) in the EquCab2 assembly. Observed base pair substitutions were used to estimate the genetic distance between the two sequences. Genetic distance was calculated as a proportion of the observed base substitutions to the total number of aligned bases excluding INDELs (p-distance). Per-base identity was estimated as a proportion of matched base pairs in the alignment to the total alignment length (including all bp in INDELs). Per-event identity was estimated as a proportion of matched base pairs in the alignment to the total number of events, where each base pair substitution and each INDEL was regarded as a single event, independent of size. We analysed variation with PipMaker dot-plot^63^ and Mauve version snapshot_2015-2-25^64^. Genomic sequence of the small structural discrepancies was compared to the genomic repetitive elements in Repbase Update database^65^.

### Investigation of heterozygous positions

Additional Sanger-sequencing was performed for five regions with dense SNP and INDEL variation at the junctions of the BAC clone CH241-147K21 with clones -455C07 and -359L18. PCR primer sequences were constructed with Primer3 and are provided in Supplementary Table S5. Amplification and sequencing was done using the BigDye Direct Cycle Sequencing Kit (Thermo Fisher Scientific) on a ProFlex PCR System (Thermo Fisher Scientific) and GeneAmp PCR System 9700 (Thermo Fisher Scientific). Genomic DNA of the horse Bravo (4 ng) was used in a 10 μl amplification reaction with an annealing temperature of 62° in accordance with the manufacturers’ protocol. Cycle sequencing was performed with both the forward (5′-TGTAAAACGACGGCCAGT-3′) and reverse (5′-CAGGAAACAGCTATGACC-3′) sequencing primers on an ABI 3500XL DNA Analyzer (Applied Biosystems) and sequences were then analysed using CodonCode Aligner v5.0.2 (CodonCode Corporation).

Heterozygosity of the 247 bp INDEL was investigated by PCR amplification and agarose gel electrophoresis. Amplification was done as described above for Sanger-sequencing and the fragment size was determined using 4% agarose gel to detect the heterozygosity of the INDEL. Genomic sequence of the INDEL was compared to the genomic repetitive elements in Repbase Update database^65^.

The genetic distance was calculated as described above for the comparison to the EquCab2 assembly and the divergence time was estimated by dividing estimated genetic distance with twice the mutation rate per site per year.

### Species comparison

Seven well annotated mammalian MHC class II regions (house mouse, human, horse, domestic dog and cat, pig and cattle) were chosen for comparative analysis. The annotation of the house mouse, human, pig, and dog was obtained from the Vega Genome Browser^66^ (release 64) and of the domestic cat and cattle from the previous publications^67^,^68^ and the RefSeq^35^ database. Alignments (Supplementary Dataset 4) were generated for mammalian coding sequences of *MHC-DRA*, -*DRB, DQA*, and -*DQB* loci using MUSCLE with the “Align Codon” option implemented in MEGA5.2.2^69^. Neighbor-Joining trees for each locus were then constructed from estimated Jukes-Cantor distances (pairwise deletion) using MEGA5.2.2. The robustness of the trees was tested by 10,000 bootstrap replications.

### Data availability

The raw sequencing data of BAC clone, LR-PCR amplicon and Sanger-sequences (Detailed information in Supplementary Table S6) can be found in European Nucleotide Archive under study number PRJEB15527 at http://www.ebi.ac.uk/ena/data/view/PRJEB15527. The final assembly and annotation of Bravo MHC class II region (LT745777) is available from the ENA browser at http://www.ebi.ac.uk/ena/data/view/LT745777.

## Additional Information

**How to cite this article:** Viļuma, A. *et al*. Genomic structure of the horse major histocompatibility complex class II region resolved using PacBio long-read sequencing technology. *Sci. Rep.* **7**, 45518; doi: 10.1038/srep45518 (2017).

**Publisher's note:** Springer Nature remains neutral with regard to jurisdictional claims in published maps and institutional affiliations.

## Supplementary Material

Supplementary Information

## Figures and Tables

**Table 1:** PacBio sequenced BAC clones from horse MHC class II minimum tiling-path.

BAC ID	Sequencing strategy	Assembled insert length (bp)	GC content (%)	Mean length of filtered subreads (bp)	Mean read coverage of the assembly
CH241-147K21	single	171,234	43.28	4207	466
CH241-455C07	single	172,837	39.40	4077	572
CH241-135M23	single	191,197	38.77	4077	387
CH241-288J19	single	205,340	39.69	3933	363
CH241-440J07	pooled*	196,174#	38.35	4533	108
CH241-359L18	pooled*	216,982	45.29	4533	120
CH241-407K07	pooled*	203,644	38.29	4600	120
CH241-441N13	pooled*	170,671	39.45	3203	287

^*^Pools of 3 BAC clones per PacBio SMRT cell.

^#^Partially assembled clone; subreads contain sequence from a single pass of a polymerase without adapter sequences.

**Table 2:** Characteristics of the overlapping clone junctions.

Junction	Component A	Component B	Overlap length (bp)	Percentage mismatch
1	CH241-407K07	CH241-288J19	54,140	0.002
2	CH241-288J19	CH241-441N13	68,481	0.002
3	CH241-441N13	CH241-440J07	99,591	0.004
4	CH241-440J07	CH241-135M23	15,669	0
5	CH241-455C07	CH241-147K21	13,475	0.23
6	CH241-147K21	CH241-359L18	141,592	0.56
7	Contig 1	Amplicon 1	122	0
8	Amplicon 1	Amplicon 2	4041	0.025
9	Amplicon 2	Contig 2	394	0

**Table 3:** BAC clone sequence differences in comparison to EquCab2 assembly.

BAC ID	No. of matched bp	No. of bp substi-tutions	No. of deleted bp (No. of events)	No. of inserted bp (No. of events)	Genetic distance* (%)	Per-base identity	Per-event identity
CH241-147K21	169,909	988	185 (78)	343 (61)	0.58	0.9912	0.9934
CH241-455C07	170,359	1785	216 (118)	214 (101)	1.04	0.9872	0.9884
CH241-135M23	190,848	230	58 (46)	49 (44)	0.12	0.9982	0.9983
CH241-288J19	205,206	64	10 (10)	16 (16)	0.03	0.9996	0.9996
CH241-440J07	212,692	1217	289 (127)	190 (113)	0.57	0.9921	0.9932
CH241-359L18	193,810	599	344 (42)	79 (45)	0.31	0.9948	0.9965
CH241-407K07	198,232	127	25 (23)	24 (24)	0.06	0.9991	0.9991
CH241-441N13	168,231	598	410 (47)	77 (44)	0.35	0.9936	0.9959

Events – number of insertions or deletions; *p-distance (nucleotide).

## References

[b1] Trowsdale J. “Both man & bird & beast”: comparative organization of MHC genes. Immunogenetics 41, 1–17 (1995). 7806269 10.1007/BF00188427

[b2] Trowsdale J. & Knight J. C. Major histocompatibility complex genomics and human disease. Annual review of genomics and human genetics 14, 301–323, doi: 10.1146/annurev-genom-091212-153455 (2013). PMC4426292 23875801

[b3] Rothbard J. B. & Gefter M. L. Interactions between immunogenic peptides and MHC proteins. Annu Rev Immunol 9, 527–565, doi: 10.1146/annurev.iy.09.040191.002523 (1991). 1910688

[b4] The MHC sequencing consortium. Complete sequence and gene map of a human major histocompatibility complex. Nature 401, 921–923, doi: http://www.nature.com/nature/journal/v401/n6756/suppinfo/401921a0_S1.html (1999). 10553908 10.1038/44853

[b5] Todd J. A. Etiology of type 1 diabetes. Immunity 32, 457–467, doi: 10.1016/j.immuni.2010.04.001 (2010). 20412756

[b6] Jones E. Y., Fugger L., Strominger J. L. & Siebold C. MHC class II proteins and disease: a structural perspective. Nature reviews. Immunology 6, 271–282, doi: 10.1038/nri1805 (2006). 16557259

[b7] Mahlios J., De la Herran-Arita A. K. & Mignot E. The autoimmune basis of narcolepsy. Current opinion in neurobiology 23, 767–773, doi: 10.1016/j.conb.2013.04.013 (2013). 23725858 PMC3848424

[b8] Fernando M. M. . Defining the role of the MHC in autoimmunity: a review and pooled analysis. PLoS genetics 4, e1000024, doi: 10.1371/journal.pgen.1000024 (2008). 18437207 PMC2291482

[b9] Hill A. V. . Molecular analysis of the association of HLA-B53 and resistance to severe malaria. Nature 360, 434–439, doi: 10.1038/360434a0 (1992). 1280333

[b10] Hill A. V. . Common west African HLA antigens are associated with protection from severe malaria. Nature 352, 595–600, doi: 10.1038/352595a0 (1991). 1865923

[b11] Schurink A. . Genome-wide association study of insect bite hypersensitivity in two horse populations in the Netherlands. Genetics Selection Evolution 44, 1–12, doi: 10.1186/1297-9686-44-31 (2012). PMC3524047 23110538

[b12] Andersson L. S. . The same ELA class II risk factors confer equine insect bite hypersensitivity in two distinct populations. Immunogenetics 64, 201–208, doi: 10.1007/s00251-011-0573-1 (2012). 21947540 PMC3276761

[b13] Klumplerova M. . Major histocompatibility complex and other allergy-related candidate genes associated with insect bite hypersensitivity in Icelandic horses. Molecular biology reports 40, 3333–3340, doi: 10.1007/s11033-012-2408-z (2013). 23275235

[b14] Staiger E. A. . Host genetic influence on papillomavirus-induced tumors in the horse. International journal of cancer, doi: 10.1002/ijc.30120 (2016). 27037728

[b15] Fritz K. L. . Genetic risk factors for insidious equine recurrent uveitis in Appaloosa horses. Animal genetics 45, 392–399, doi: 10.1111/age.12129 (2014). 24467435

[b16] Ansari H. A., Hediger R., Fries R. & Stranzinger G. Chromosomal localization of the major histocompatibility complex of the horse (ELA) by *in situ* hybridization. Immunogenetics 28, 362–364 (1988). 3169882 10.1007/BF00364235

[b17] Albright-Fraser D., Reid R., Gerber V. & Bailey E. Polymorphism of DRA among equids. Immunogenetics 43, 315–317, doi: 10.1007/BF02440999 (1996). 9110935

[b18] Fraser D. G. & Bailey E. Polymorphism and multiple loci for the horse DQA gene. Immunogenetics 47, 487–490, doi: 10.1007/s002510050387 (1998). 9553156

[b19] Brown J. J. . Polymorphisms of the equine major histocompatibility complex class II DRA locus. Tissue antigens 64, 173–179, doi: 10.1111/j.1399-0039.2004.00269.x (2004). 15245372

[b20] Diaz S., Giovambattista G., Dulout F. N. & Peral-Garcia P. Genetic variation of the second exon of ELA-DRB genes in Argentine Creole horses. Animal genetics 32, 257–263 (2001). 11683711 10.1046/j.1365-2052.2001.00779.x

[b21] Gustafsson K. & Andersson L. Structure and polymorphism of horse MHC class II DRB genes: convergent evolution in the antigen binding site. Immunogenetics 39, 355–358 (1994). 8168853 10.1007/BF00189233

[b22] Wade C. M. . Genome sequence, comparative analysis, and population genetics of the domestic horse. Science 326, 865–867, doi: 10.1126/science.1178158 (2009). 19892987 PMC3785132

[b23] Osoegawa K. . An improved approach for construction of bacterial artificial chromosome libraries. Genomics 52, 1–8, doi: 10.1006/geno.1998.5423 (1998). 9740665

[b24] Tallmadge R. L., Lear T. L. & Antczak D. F. Genomic characterization of MHC class I genes of the horse. Immunogenetics 57, 763–774, doi: 10.1007/s00251-005-0034-9 (2005). 16220348

[b25] Gustafson A. L. . An ordered BAC contig map of the equine major histocompatibility complex. Cytogenetic and Genome Research 102, 189–195, doi: 10.1159/000075747 (2003). 14970701

[b26] Vij S. . Chromosomal-Level Assembly of the Asian Seabass Genome Using Long Sequence Reads and Multi-layered Scaffolding. PLoS genetics 12, e1005954, doi: 10.1371/journal.pgen.1005954 (2016). 27082250 PMC4833346

[b27] Frank J. A. . Improved metagenome assemblies and taxonomic binning using long-read circular consensus sequence data. Scientific reports 6, 25373, doi: 10.1038/srep25373 (2016). 27156482 PMC4860591

[b28] Huddleston J. . Reconstructing complex regions of genomes using long-read sequencing technology. Genome research 24, 688–696, doi: 10.1101/gr.168450.113 (2014). 24418700 PMC3975067

[b29] Chaisson M. J. . Resolving the complexity of the human genome using single-molecule sequencing. Nature 517, 608–611, doi: 10.1038/nature13907 (2015). 25383537 PMC4317254

[b30] Santagostino M. . Genome-wide evolutionary and functional analysis of the Equine Repetitive Element 1: an insertion in the myostatin promoter affects gene expression. BMC genetics 16, 126, doi: 10.1186/s12863-015-0281-1 (2015). 26503543 PMC4623272

[b31] Orlando L. . Recalibrating Equus evolution using the genome sequence of an early Middle Pleistocene horse. Nature 499, 74–78, doi: 10.1038/nature12323 (2013). 23803765

[b32] Bergstrom T. F., Josefsson A., Erlich H. A. & Gyllensten U. Recent origin of HLA-DRB1 alleles and implications for human evolution. Nature genetics 18, 237–242, doi: 10.1038/ng0398-237 (1998). 9500545

[b33] Hestand M. S. . Annotation of the Protein Coding Regions of the Equine Genome. PLoS One 10, e0124375, doi: 10.1371/journal.pone.0124375 (2015). 26107351 PMC4481266

[b34] Maccari G. . IPD-MHC 2.0: an improved inter-species database for the study of the major histocompatibility complex. Nucleic acids research 45, D860–D864, doi: 10.1093/nar/gkw1050 (2017). 27899604 PMC5210539

[b35] O’Leary N. A. . Reference sequence (RefSeq) database at NCBI: current status, taxonomic expansion, and functional annotation. Nucleic acids research 44, D733–745, doi: 10.1093/nar/gkv1189 (2016). 26553804 PMC4702849

[b36] Murphy W. J., Pringle T. H., Crider T. A., Springer M. S. & Miller W. Using genomic data to unravel the root of the placental mammal phylogeny. Genome research 17, 413–421, doi: 10.1101/gr.5918807 (2007). 17322288 PMC1832088

[b37] Rhoads A. & Au K. F. PacBio Sequencing and Its Applications. Genomics, Proteomics & Bioinformatics 13, 278–289, doi: 10.1016/j.gpb.2015.08.002 (2015). PMC4678779 26542840

[b38] Chin C.-S. . Nonhybrid, finished microbial genome assemblies from long-read SMRT sequencing data. Nat Meth 10, 563–569, doi: 10.1038/nmeth.2474 http://www.nature.com/nmeth/journal/v10/n6/abs/nmeth.2474.html#supplementary-information (2013). 23644548

[b39] Korlach J. Understanding Accuracy in SMRT^®^ Sequencing, http://www.pacb.com/wp-content/uploads/2015/09/Perspective_UnderstandingAccuracySMRTSequencing1.pdf (2013).

[b40] Pacholewska A. . The transcriptome of equine peripheral blood mononuclear cells. PLoS One 10, e0122011, doi: 10.1371/journal.pone.0122011 (2015). 25790166 PMC4366165

[b41] Fraser D. G. & Bailey E. Demonstration of threeDRB locl in a domestic horse family. Immunogenetics 44, 441–445, doi: 10.1007/BF02602805 (1996). 8824155

[b42] Sutton V. R. & Knowles R. W. An aberrant DRB4 null gene transcript is found that could encode a novel HLA-DR beta chain. Immunogenetics 31, 112–117 (1990). 2303277 10.1007/BF00661221

[b43] Horin P. & Matiasovic J. A second locus and new alleles in the major histocompatibility complex class II (ELA-DQB) region in the horse. Animal genetics 33, 196–200 (2002). 12030922 10.1046/j.1365-2052.2002.00839.x

[b44] Rask L., Jonsson A. K., Svensson A. C., Gustafsson K. & Andersson L. The structure of human MHC class II genes. Autoimmunity 8, 237–244 (1991). 1932510 10.3109/08916939108997111

[b45] Antczak D. F. A life with horses: it’s been a great ride! Vet Immunol Immunopathol 148, 6–11, doi: 10.1016/j.vetimm.2012.05.015 (2012). 22673194

[b46] McCue M. E. . A high density SNP array for the domestic horse and extant Perissodactyla: utility for association mapping, genetic diversity, and phylogeny studies. PLoS genetics 8, e1002451, doi: 10.1371/journal.pgen.1002451 (2012). 22253606 PMC3257288

[b47] Bodmer J. G. . Nomenclature for factors of the HLA system, 1991. International journal of immunogenetics 19, 95–119, doi: 10.1111/j.1744-313X.1992.tb00050.x (1992). 1627537

[b48] Andersson G. . Class II genes of the human major histocompatibility complex. Organization and evolutionary relationship of the DR beta genes. The Journal of biological chemistry 262, 8748–8758 (1987). 3036826

[b49] Kelley J., Walter L. & Trowsdale J. Comparative genomics of major histocompatibility complexes. Immunogenetics 56, 683–695, doi: 10.1007/s00251-004-0717-7 (2005). 15605248

[b50] Nei M., Gu X. & Sitnikova T. Evolution by the birth-and-death process in multigene families of the vertebrate immune system. Proceedings of the National Academy of Sciences of the United States of America 94, 7799–7806 (1997). 9223266 10.1073/pnas.94.15.7799 PMC33709

[b51] Chin C. S. . Nonhybrid, finished microbial genome assemblies from long-read SMRT sequencing data. Nature methods 10, 563–569, doi: 10.1038/nmeth.2474 (2013). 23644548

[b52] Li H. Aligning sequence reads, clone sequences and assembly contigs with BWA-MEM. *ArXiv e-prints* (2013).

[b53] Li H. . The Sequence Alignment/Map format and SAMtools. Bioinformatics (Oxford, England) 25, 2078–2079, doi: 10.1093/bioinformatics/btp352 (2009). PMC2723002 19505943

[b54] Quinlan A. R. & Hall I. M. BEDTools: a flexible suite of utilities for comparing genomic features. Bioinformatics (Oxford, England) 26, 841–842, doi: 10.1093/bioinformatics/btq033 (2010). PMC2832824 20110278

[b55] Bonfield J. K. & Whitwham A. Gap5—editing the billion fragment sequence assembly. Bioinformatics (Oxford, England) 26, 1699–1703, doi: 10.1093/bioinformatics/btq268 (2010). PMC2894512 20513662

[b56] Untergasser A. . Primer3–new capabilities and interfaces. Nucleic acids research 40, e115, doi: 10.1093/nar/gks596 (2012). 22730293 PMC3424584

[b57] Berlin K. . Corrigendum: Assembling large genomes with single-molecule sequencing and locality-sensitive hashing. Nature biotechnology 33, 1109, doi: 10.1038/nbt1015-1109c (2015). 26448093

[b58] Smit A. F. A., Hubley R. & Green P. RepeatMasker Web Server. *unpublished data. Current Version: open-4.0.5 (RMLib: 20140131 & Dfam: 1.3*) (2014).

[b59] Stanke M. & Waack S. Gene prediction with a hidden Markov model and a new intron submodel. Bioinformatics (Oxford, England) 19 Suppl 2, ii215–225 (2003). 10.1093/bioinformatics/btg1080 14534192

[b60] Laurens Wilming A. F., Loveland Jane, Mudge Jonathan & Steward Charles, Jennifer Harrow, HAVANA team. HAVANA annotation guidelines. 48 (2012).

[b61] Misra S. . Annotation of the Drosophila melanogaster euchromatic genome: a systematic review. Genome biology 3, RESEARCH0083 (2002). 10.1186/gb-2002-3-12-research0083 PMC151185 12537572

[b62] Chaisson M. J. & Tesler G. Mapping single molecule sequencing reads using basic local alignment with successive refinement (BLASR): application and theory. BMC bioinformatics 13, 238, doi: 10.1186/1471-2105-13-238 (2012). 22988817 PMC3572422

[b63] Schwartz S. . PipMaker–a web server for aligning two genomic DNA sequences. Genome research 10, 577–586 (2000). 10779500 10.1101/gr.10.4.577 PMC310868

[b64] Darling A. E., Mau B. & Perna N. T. ProgressiveMauve: multiple genome alignment with gene gain, loss and rearrangement. PLoS One 5, e11147, doi: 10.1371/journal.pone.0011147 (2010). 20593022 PMC2892488

[b65] Jurka J. Repbase Update: a database and an electronic journal of repetitive elements. Trends in Genetics 16, 418–420, doi: http://dx.doi.org/10.1016/S0168-9525(00)02093-X (2000). 10973072 10.1016/s0168-9525(00)02093-x

[b66] Wilming L. G. . The vertebrate genome annotation (Vega) database. Nucleic acids research 36, D753–D760, doi: 10.1093/nar/gkm987 (2008). 18003653 PMC2238886

[b67] Yuhki N. . Comparative genome organization of human, murine, and feline MHC class II region. Genome research 13, 1169–1179, doi: 10.1101/gr.976103 (2003). 12743023 PMC403645

[b68] Childers C. P. . Comparative analysis of the bovine MHC class IIb sequence identifies inversion breakpoints and three unexpected genes. Animal genetics 37, 121–129, doi: 10.1111/j.1365-2052.2005.01395.x (2006). 16573526

[b69] Tamura K. . MEGA5: Molecular Evolutionary Genetics Analysis Using Maximum Likelihood, Evolutionary Distance, and Maximum Parsimony Methods. Molecular Biology and Evolution 28, 2731–2739, doi: 10.1093/molbev/msr121 (2011). 21546353 PMC3203626

[b70] Ellis S. A. . ISAG/IUIS-VIC Comparative MHC Nomenclature Committee report, 2005. Immunogenetics 57, 953–958, doi: 10.1007/s00251-005-0071-4 (2006). 16389556

