# Supplementary material for: Genomic structure of the horse major histocompatibility complex class II region resolved using PacBio long-read sequencing technology
Source: Sci Rep. 2017 Mar 31;7:45518. doi: 10.1038/srep45518 (PMC5374520; doi:10.1038/srep45518)
Supplement: Supplementary Information [file srep45518-s1.pdf]

Genomic structure of the horse major histocompatibility complex class II region resolved using PacBio long-read sequencing technology

Agnese Viļuma, Sofia Mikko, Daniela Hahn, Loren Skow, Göran Andersson, Tomas F. Bergström

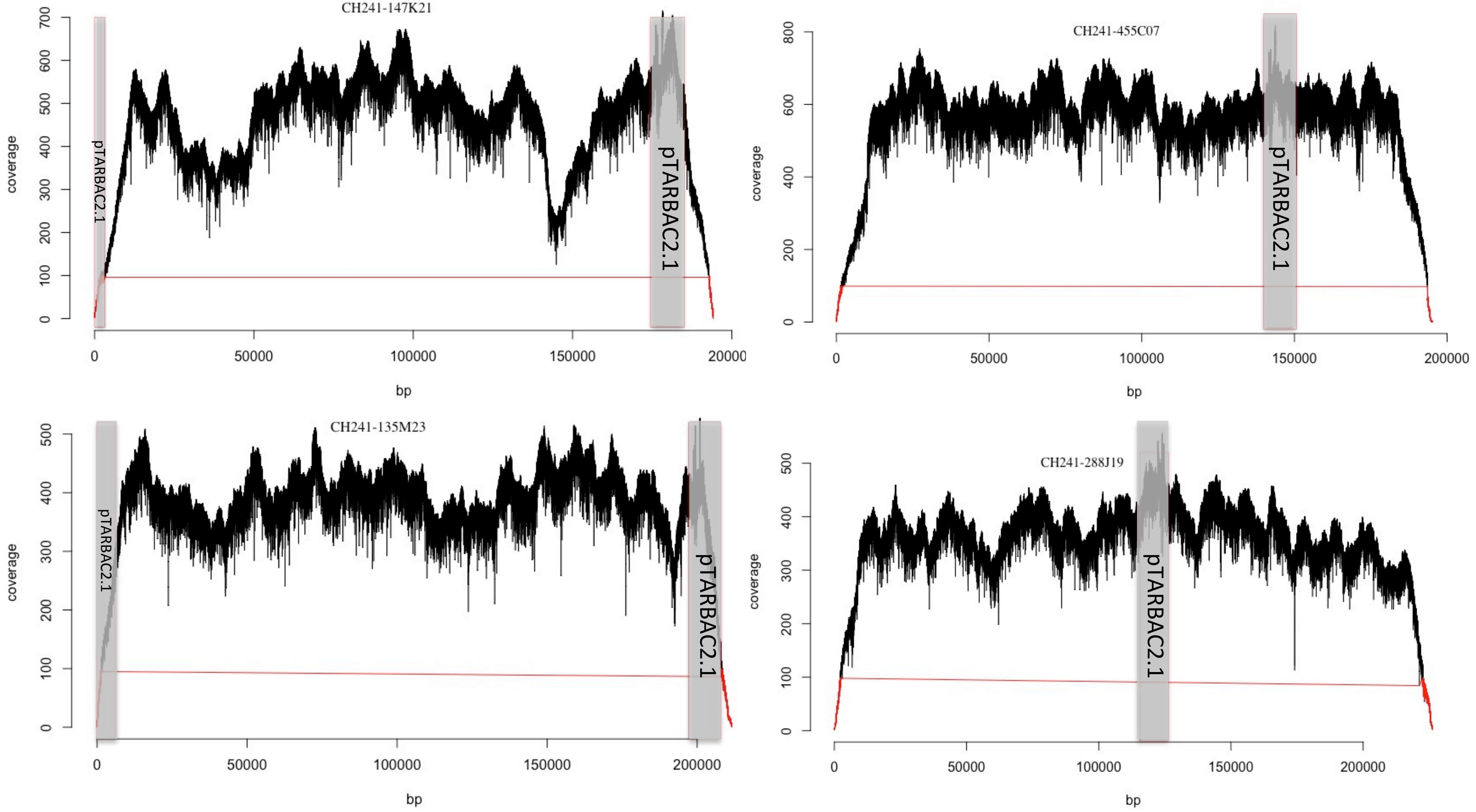

Supplementary Figure S1. Coverage of each randomly linearized BAC clone construct assembly, where the position(s) of the pTARBAC2.1 vector is shown as a grey box. Coverage describes the number of filtered subreads supporting each base pair of the final assembly. The threshold for end trimming is indicated as a red vertical line.

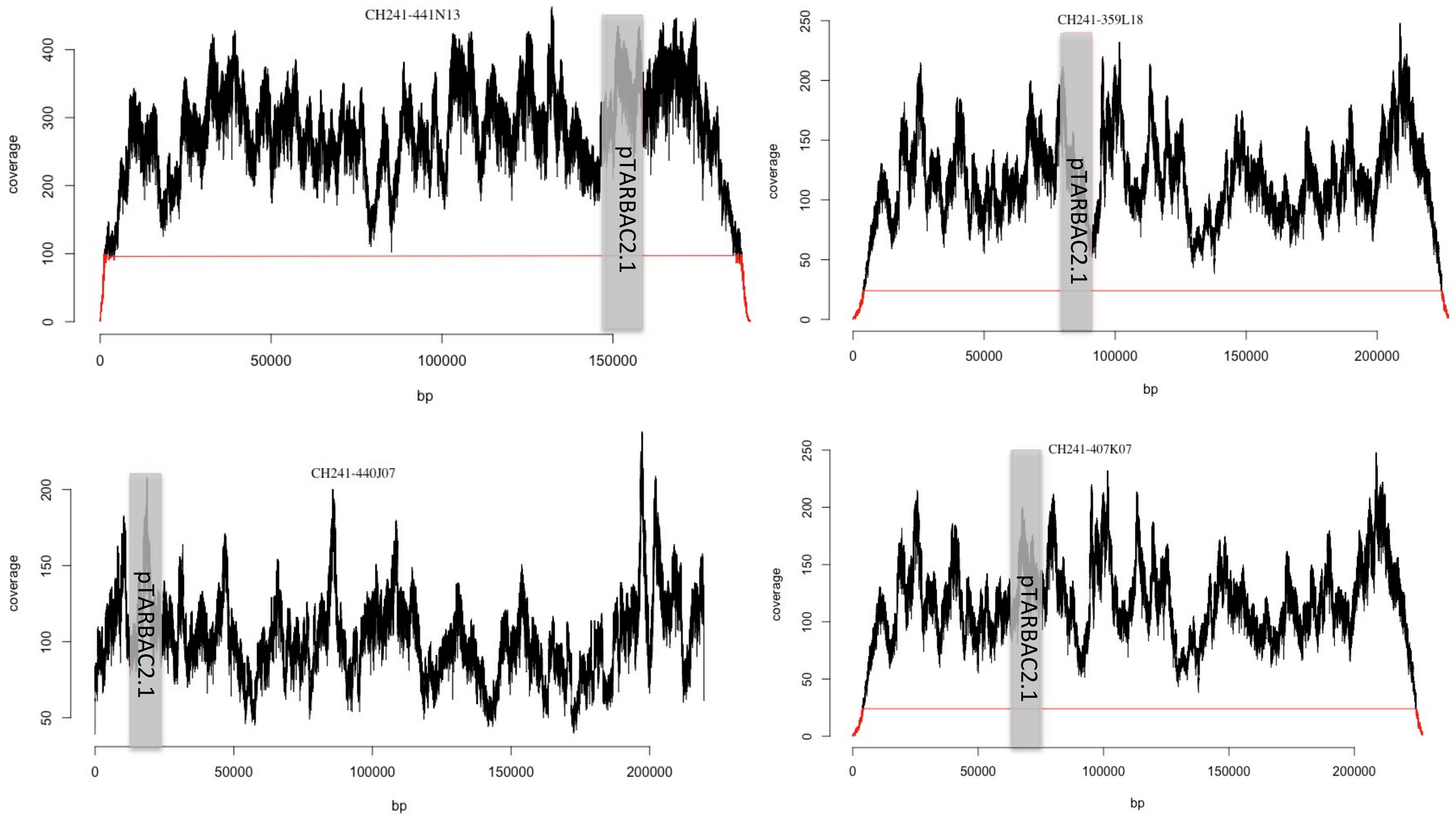

Supplementary Figure S1 continued.

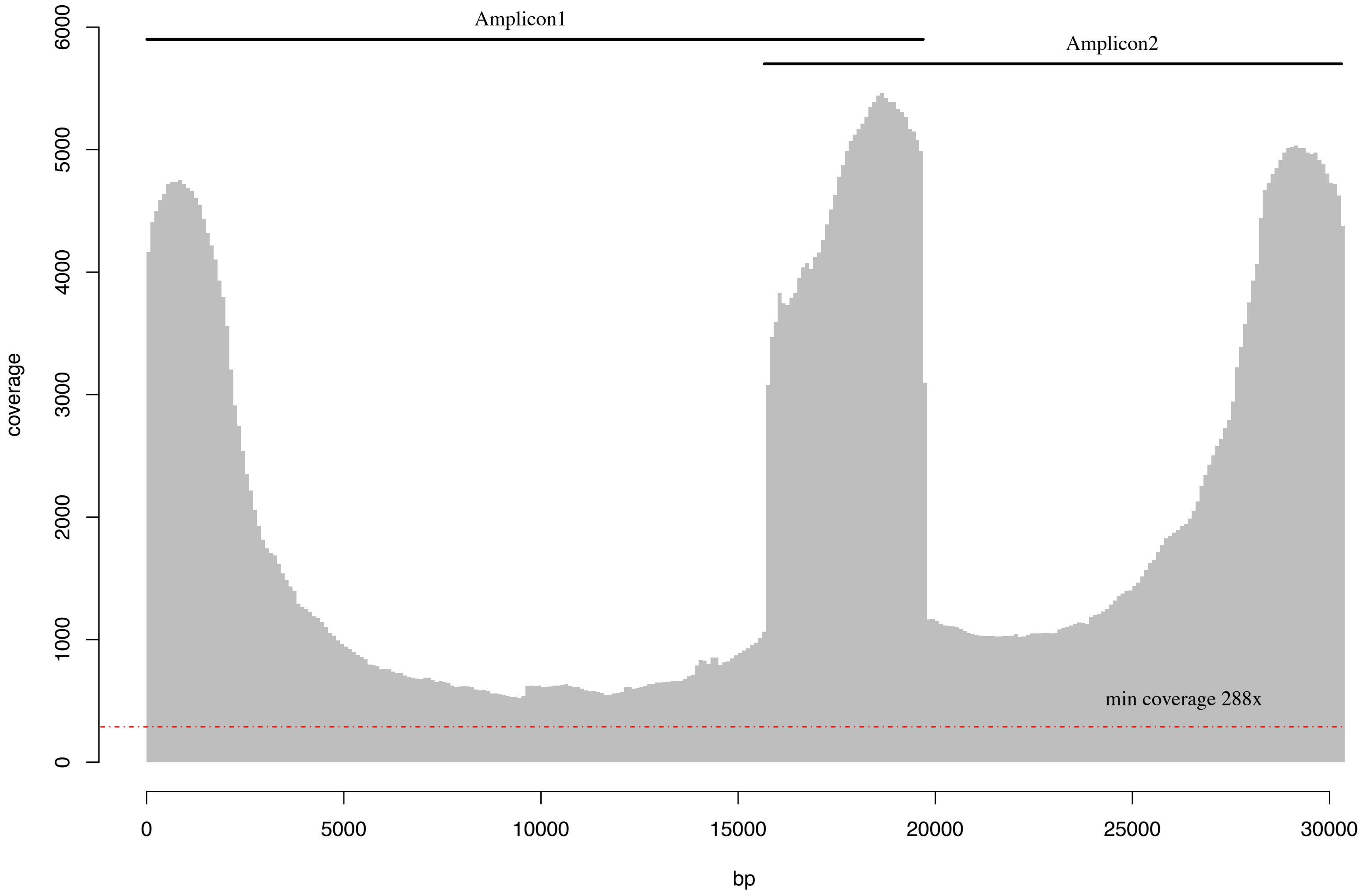

Supplementary Figure S2. Overview of the two long-range PCR amplicon coverage. Coverage describes the number of reads of insert covering each base pair of the final amplicon assembly. Red dotted line indicates the minimum observed per base coverage.

```
CLUSTAL O(1.2.3) multiple sequence alignment

Eqca-DQB3                 MSGKMAPQIPRGLWTAAMMAMLVVLSTPVAEGRDSPQDFVFQLKGQCYFTNGTERVRLVTRLIYNREEFVRFDSDVGEFQAVTELGRHIAEDWNGQKDVLEQKRAELDTVCRHNYQVEAL
Eqca-DQB1                 MSGKMAPQMPRGLWTAAVMVTLVVLSIPVAEGRDSPKDFVIQLKGLCYFTNGTERVRLVTRYIYNREEWVRFDSDVGEYRALTEQGRPDAEDWNGQKDFLEQTRAYVDTVCRHNYQVEAP
Eqca-DQB2                 ----MAPQIPRGLWTAAVMVMLVVLSTPVAEGRDSPQDFVVQFMGQCYFTNGTEHVRYVTRYIYNREEYVRFDSDVGEYRALTELGRPEAEYWNGQKDILEGTRAELDRVCRPNYQLEFP
sp|P01920|DQB1_HUMAN      MSWKKALRIPGGLRAATVTLMLAMLSTPVAEGRDSPEDFVYQFKAMCYFTNGTERVRYVTRYIYNREEYARFDSDVEVYRAVTPLGPPDAEYWNSQKEVLERTRAELDTVCRHNYQLELR
sp|P05538|DQB2_HUMAN      MSWKMALQIPGGFWAAAVTVMLVMLSTPVAEARDFPKDFLVQFKGMCYFTNGTERVRGVARYIYNREEYGRFDSDVGEFQAVTELGRS-IEDWNNYKDFLEQERAAVDKVCRHNYEAELR
                               * ::* *: :*::   *.:** ****.** *:**: *: . ********:** *:* ******: ******  ::*:*  *    * **. *:.**  ** :* *** **: *
                          Exon 1                                      Exon 2

Eqca-DQB3                 TTLQRRVEPRVTISPSRTEVLNRHNLLVCSVTDFYPGQIKVRWFRNDQEETAGIVSTPLIRNGDWTFQILVMLEMTPQRGDVYTCHVEHPSPQSPITVQWRTHSESAQNKMLCGIGSFVL
Eqca-DQB1                 FTWQRRVKPTVTISPSRTEALNHHNLLVCSVTDFYPGQIKVQWFRNDQEETAGVVSTPLIRNGDWTFQILVMLEMTPQRGDVYTCHVEHPSLQSPITVQWQVQSESAQSKMLSGIGGFVL
Eqca-DQB2                 A-LERRVEPRVTISPSRTEALNHHNLLVCSVTDFYPGQIKVRWFRNGQEETAGVVSTPLIRNGDWTFQILVMLEMTPQRGDVYTCHVEHPSLQSPITVEWQAQSESAQSKMLSGVGGFML
sp|P01920|DQB1_HUMAN      TTLQRRVEPTVTISPSRTEALNHHNLLVCSVTDFYPAQIKVRWFRNDQEETTGVVSTPLIRNGDWTFQILVMLEMTPQHGDVYTCHVEHPSLQNPITVEWRAQSESAQSKMLSGIGGFVL
sp|P05538|DQB2_HUMAN      TTLQRQVEPTVTISPSRTEALNHHNLLVCSVTDFYPAQIKVRWFRNDQEETAGVVSTSLIRNGDWTFQILVMLEITPQRGDIYTCQVEHPSLQSPITVEWRAQSESAQSKMLSGIGGFVL
                             :*:*:* *********.**:*************.****:****.****:*:*** ***************:***:**:***:***** *.****:*:.:*****.***.*:*.*:*
                                                           Exon 3                                                          Exon 4

Eqca-DQB3                 GLVFLGLSLIIHHRSQKGPRGSPPADCIREAH
Eqca-DQB1                 GLIFLGLGLIIHHRSKKGPRGPSPAGLLD---
Eqca-DQB2                 GLIFLGLGLIIHHRSQKGPRGPPPAGLLR---
sp|P01920|DQB1_HUMAN      GLIFLGLGLIIHHRSQK--------GLLH---
sp|P05538|DQB2_HUMAN      GLIFLGLGLIIRHRGQKGPRGPPPAGLLH---
                          **:****.***:**.:**
                                           Exon 5 Exon 6
```

Supplementary Figure S3. Multiple alignment (ClustalO, http://www.ebi.ac.uk/Tools/msa/clustalo/) of the predicted protein sequences of the three functional *Eqca-DQB* genes and *HLA-DQB1* (P01920) and *-DQB2* (P05538). The amino acid sequence of the *Eqca-DQB3* exon 6 is highlighted in red.

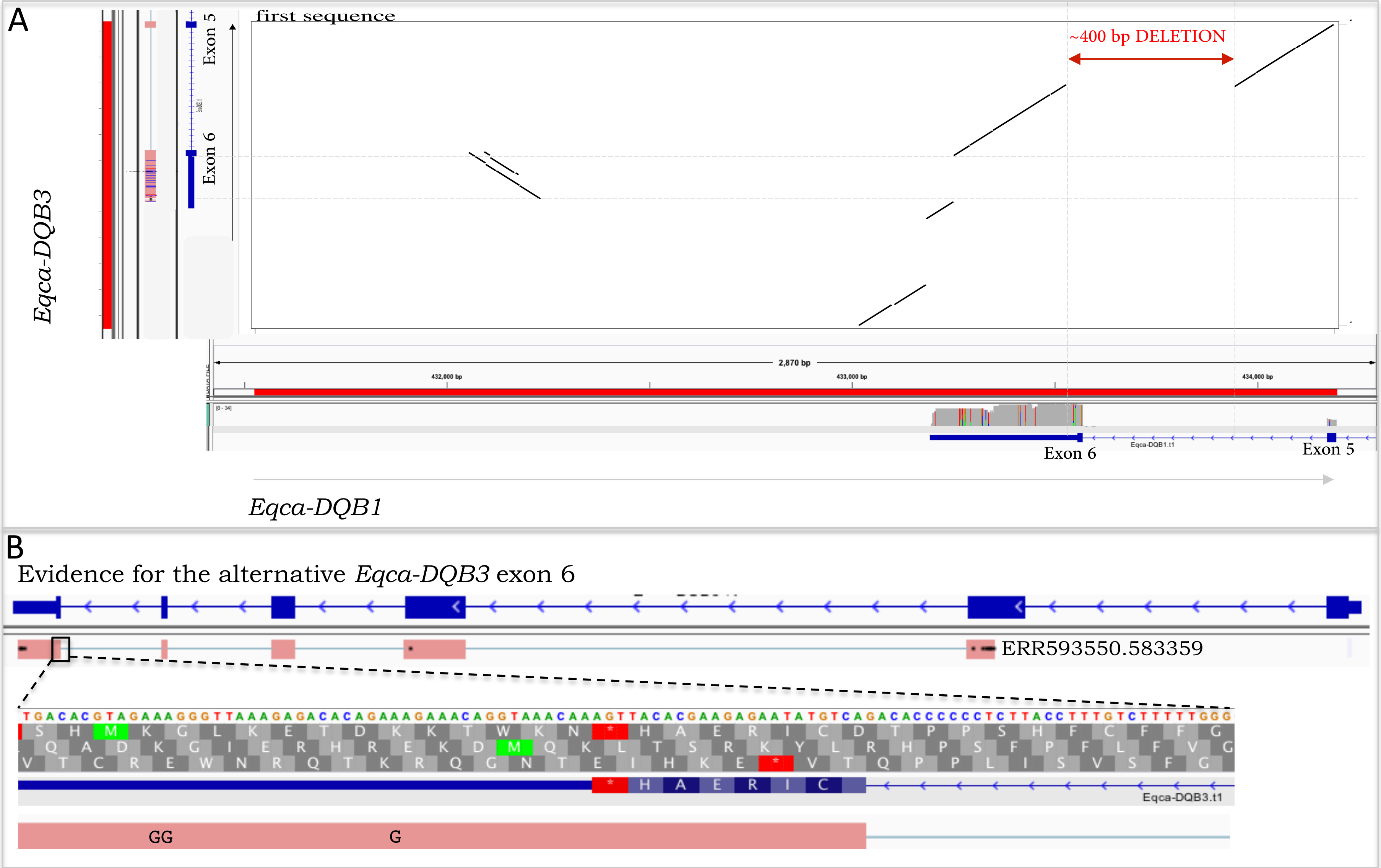

Supplementary Figure S4. A)Dot-plot comparison of the *Eqca-DQB3* and *Eqca-DQB1* genomic sequence. The 400 bp deletion of the *Eqca-DQB3* sequence spans a portion of intron 5 and the entire protein coding sequence of exon 6 of *Eqca-DQB1*. The annotation tracks (in blue) are displayed below (*Eqca-DQB1*) and on the left side (*Eqca-DQB3*) of the dot-plot. B) The RNA-seq read (Roche 454) supporting the alternative exon 6 of *Eqca-DQB3* (in pink).

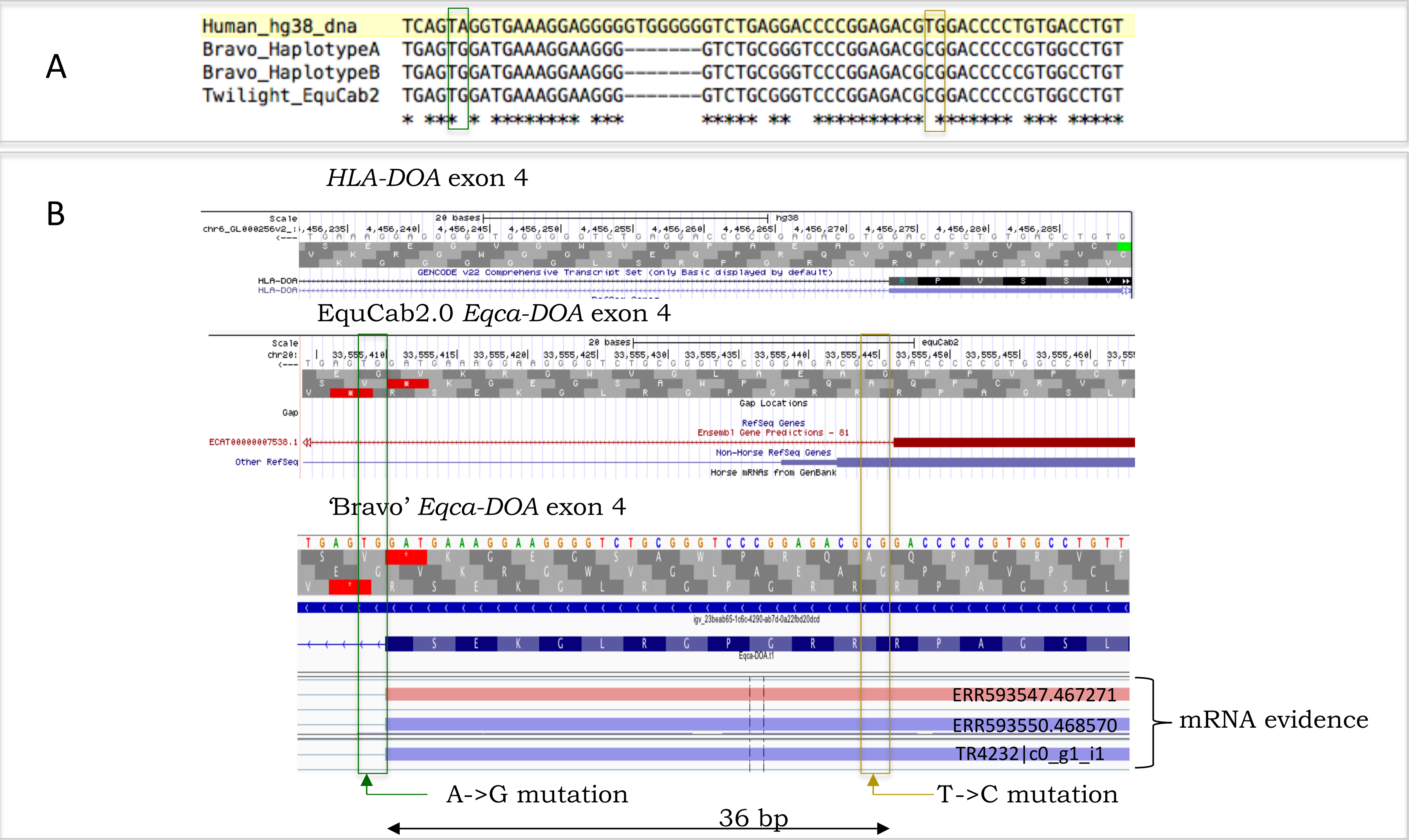

Supplementary Figure S5. Figure illustrates the alternative donor splice site of the *Eqca-DOA* exon 4. A) Multiple sequence alignment of coding sequences at the 3' end of exon 4 and 5' end of intron 5 of the *MHC-DOA* gene in human, Bravo haplotypes and EquCab2 assembly. The human splice site mutation and the alternative canonical splice site of the horse are highlighted with yellow and green boxes, respectively. B) Annotation tracks from the UCSC genome browser (*HLA-DOA* and EquCab2 *Eqca-DOA*) and from the IGV visualization tool (Bravo *Eqca-DOA*) illustrate the current annotation of these genes. Aligned mRNA evidence supporting the alternative canonical splice site of the extended exon 4 is shown in contrast to the RefSeq Genes and Ensembl Gene Predictions.

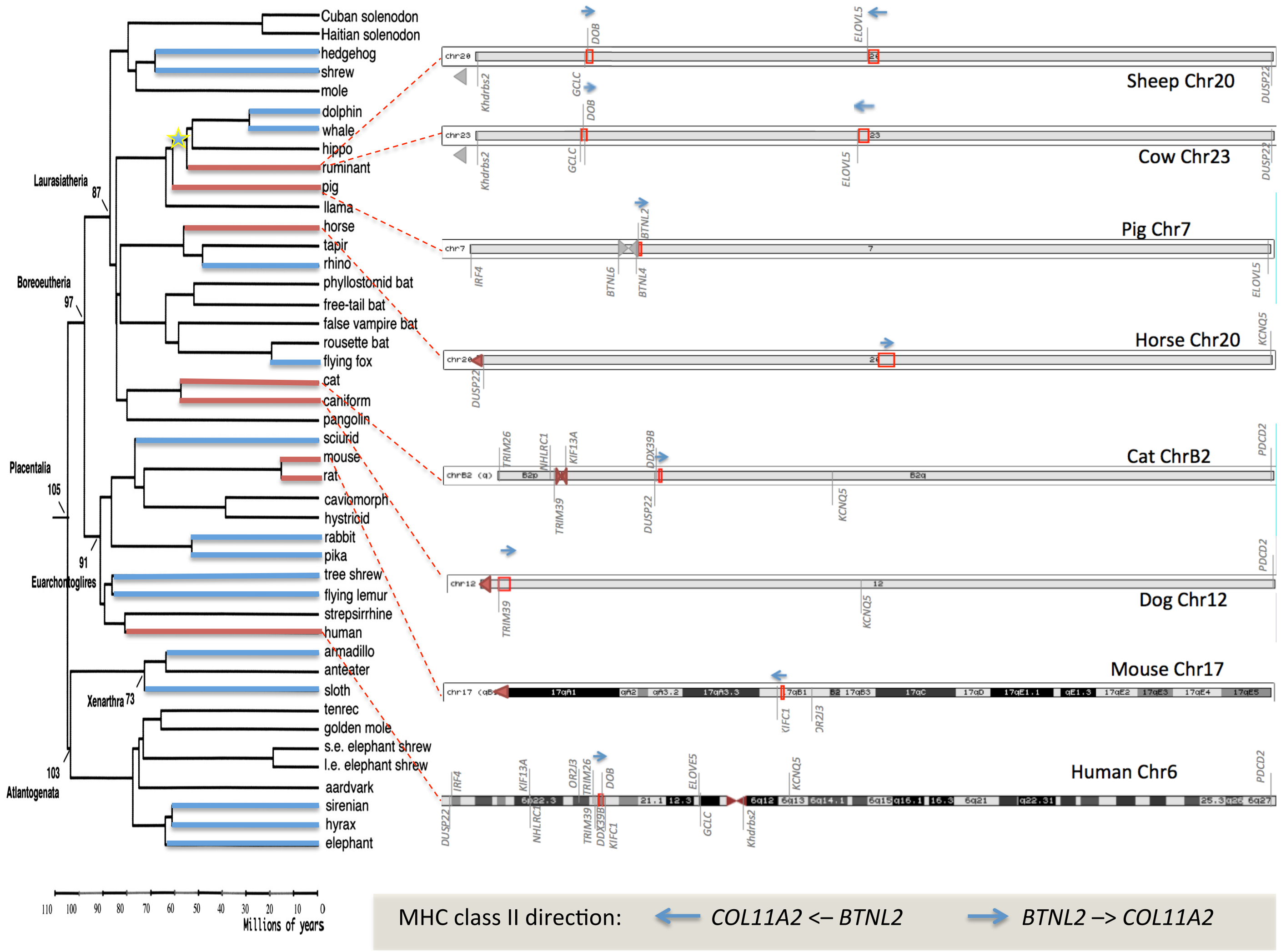

Supplementary Figure S6. This figure illustrates our choice of species for comparative analysis according to their evolutionary distance, genome and annotation completeness. Phylogenetic branches in blue represent species where genome assemblies are present as separate scaffolds and/or lack proper annotation. Phylogenetic branches in red represent species with a reliable MHC class II assembly and annotation (e.g. specific publications, *RefSeq* gene annotation). Chromosomal location of the MHC class II region is indicated with a red box and the directionality is shown with a blue arrow. For ruminants, two well annotated sequences of cattle and sheep MHC class II region are available. Both of these sequences share a characteristic of the split MHC class II region due to an ancestral inversion (indicated with a star). In our species comparison (Figure 4), we retained cattle as a representative of the ruminants.

<u>Phylogenetic tree from:</u> Murphy, W. J., Pringle, T. H., Crider, T. A., Springer, M. S. & Miller, W. Using genomic data to unravel the root of the placental mammal phylogeny. *Genome research* **17**, 413-421, doi:10.1101/gr.5918807 (2007).

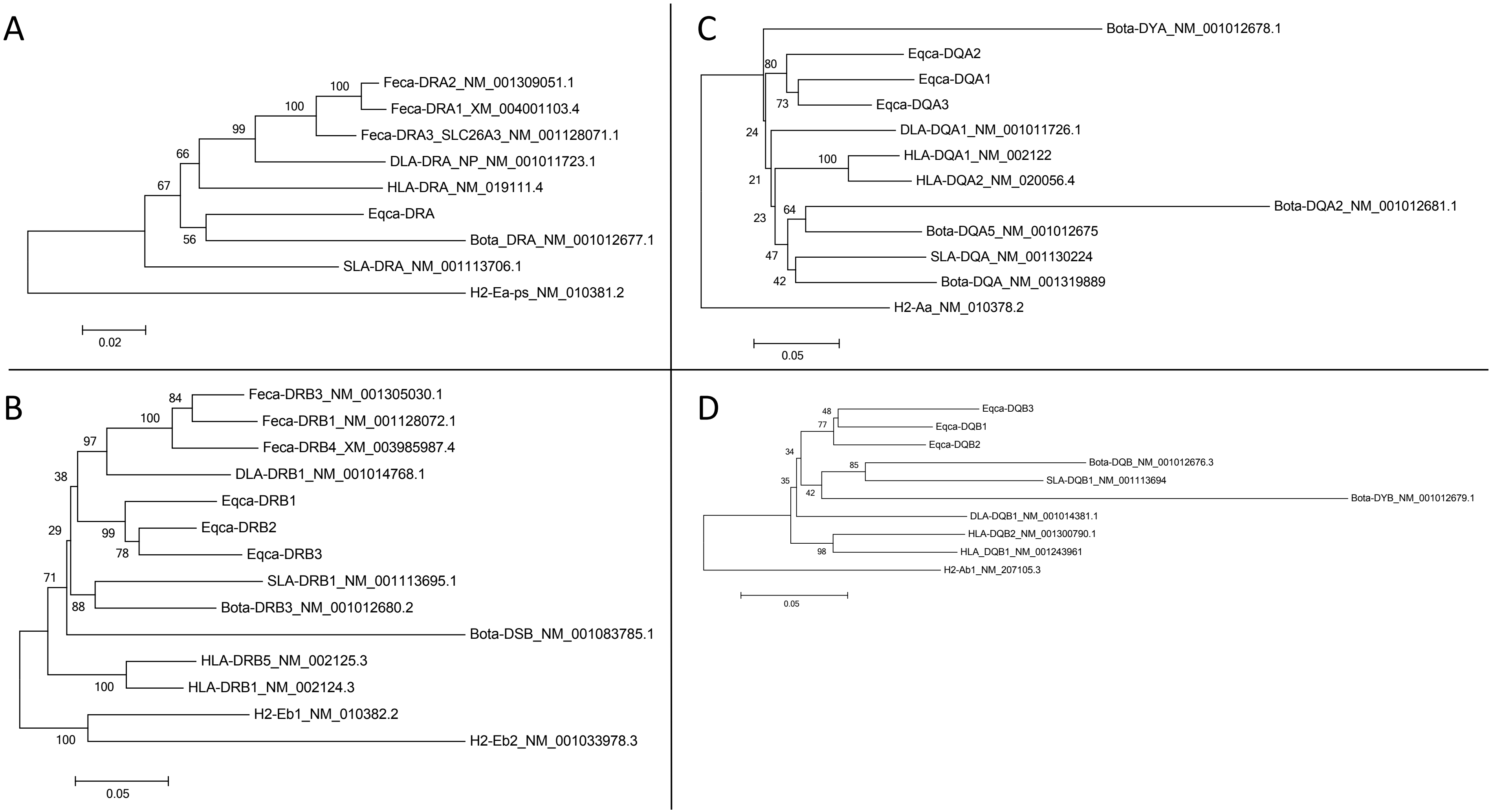

Supplementary Figure S7. Phylogenetic relationship of seven mammalian *MHC-DRA*, *-DRB*, *-DQA* and *DQB* loci are illustrated in the sections A, B, C and D, respectively. Neighbor-Joining trees were constructed from estimated Jukes-Cantor distances (pairwise deletion) of CDS and the robustness of the branching order was estimated by 10,000 bootstrap replications. Gene names are followed by a RefSeq accession number for each sequence.

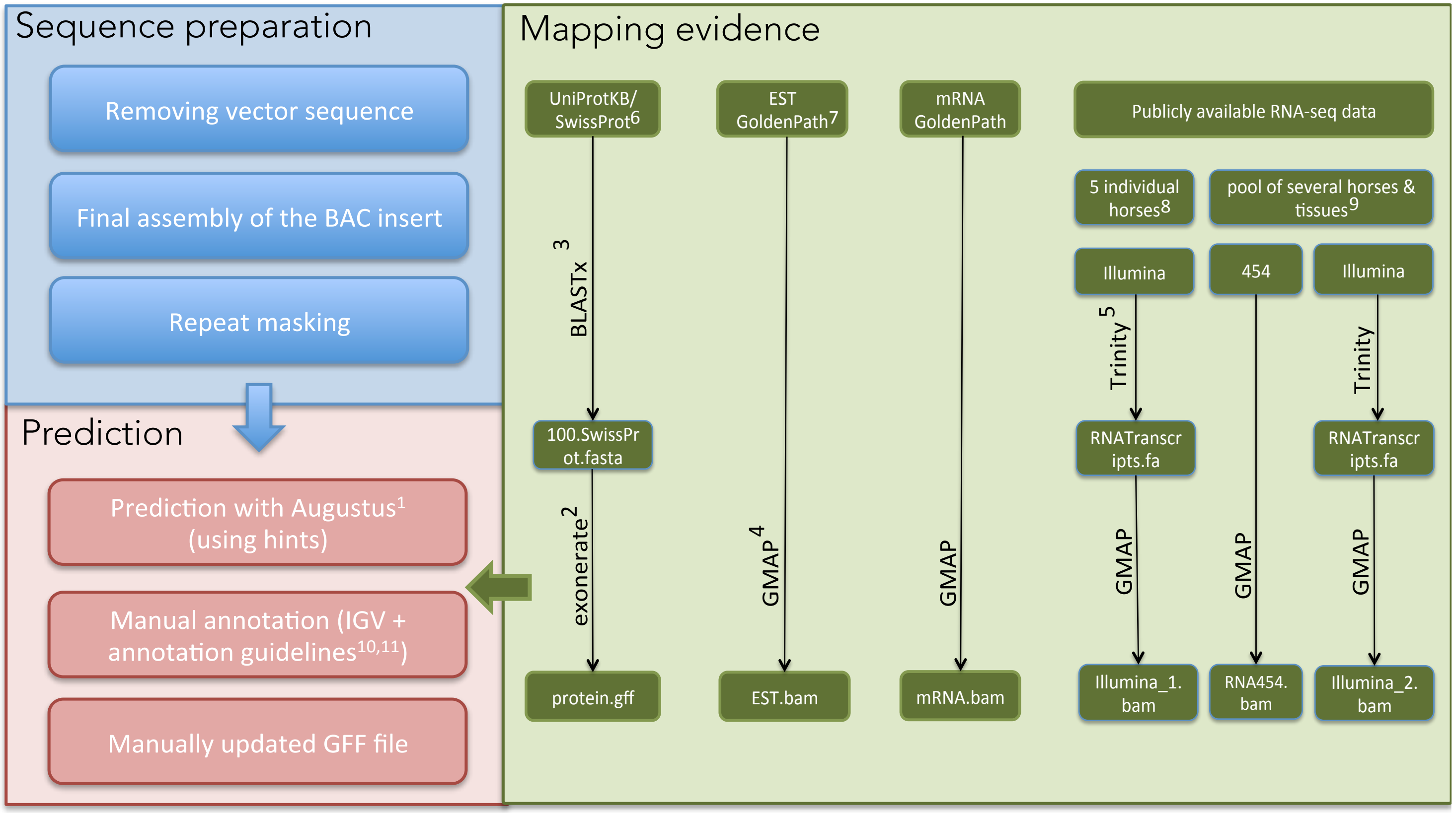

Supplementary Figure S8. A schematic overview of the automated annotation pipeline and tools involved.

1. Stanke, M. & Waack, S. Gene prediction with a hidden Markov model and a new intron submodel. *Bioinformatics (Oxford, England)* **19 Suppl 2**, ii215-225 (2003).
2. Slater, G. S. C. & Birney, E. Automated generation of heuristics for biological sequence comparison. BMC bioinformatics 6, 1-11, doi:10.1186/1471-2105-6-31 (2005).
3. http://blast.ncbi.nlm.nih.gov/Blast.cgi
4. Wu, T. D., Reeder, J., Lawrence, M., Becker, G. & Brauer, M. J. GMAP and GSNAP for Genomic Sequence Alignment: Enhancements to Speed, Accuracy, and Functionality. Methods in molecular biology (Clifton, N.J.) 1418, 283-334, doi:10.1007/978-1-4939-3578-9_15 (2016).
5. Slater, G. S. C. & Birney, E. Automated generation of heuristics for biological sequence comparison. BMC bioinformatics 6, 1-11, doi:10.1186/1471-2105-6-31 (2005).
6. Consortium, T. U. UniProt: a hub for protein information. Nucleic acids research 43, D204-D212, doi:10.1093/nar/gku989 (2015).
7. http://hgdownload.cse.ucsc.edu/downloads.html
8. Hestand, M. S. et al. Annotation of the Protein Coding Regions of the Equine Genome. PLoS One 10, e0124375, doi:10.1371/journal.pone.0124375 (2015).
9. Pacholewska, A. et al. The transcriptome of equine peripheral blood mononuclear cells. PLoS One 10, e0122011, doi:10.1371/journal.pone.0122011 (2015).
10. Laurens Wilming, A. F., Jane Loveland, Jonathan Mudge, Charles Steward, Jennifer Harrow, HAVANA team. HAVANA annotation guidelines. 48 (2012).
11. Misra, S. et al. Annotation of the Drosophila melanogaster euchromatic genome: a systematic review. Genome biology 3, RESEARCH0083 (2002).

Supplementary Table S1. Genotyping results of the SNP and INDEL dense regions observed in junctions five and six.

		Expected*		Confirmed		
Region No.	Genomic location in MHC class II	SNPs	INDELs	SNPs	INDELs	Method
1	926,154-926,727	4	1	4	0	Sanger-seq
2	957,974-958,934	16	2	16	2	Sanger-seq
3	1,012,387-1,013,364	10	1	9	1	Sanger-seq
4	1,066,875-1,067,709	7	0	7	0	Sanger-seq
5	1,088,221-1,089,269	9	0	9	0	Sanger-seq
247 bp INDEL	1,016,235-1,017,082	0	1	0	1	4% agarose gel
	**Sum:**	**46**	**5**	**45**	**4**	

*Expected number of SNPs and INDELs based on PacBio sequencing of the BAC clones

Supplementary Table S2. Summary of the long-range PCR amplicon sequencing results.

	Expected length (bp)	Observed length (bp)
Amplicon 1	16,512	19,701
Amplicon 2	12,953	14,649
Assembly of Amplicon 1 and 2	28,151	30,309
Amplicon 1 and 2 overlap	1,314	4,041
CH241-135M23 and Amplicon 1 overlap	122	122
CH241-455C07 and Amplicon 2 overlap	394	394

Supplementary Table S3. Gene content of the BAC clone sequences.

BAC ID	No. of genes	Genes	No. of pseudo-genes	Pseudogenes
CH241-407K07	3	*LOC504295*, *LOC525599*, *BTNL2*	1	*Eqca-G*
CH241-288J19	4	*BTNL2**, *Eqca-DRA*, *-DRB1*, *-DQA1*	2	*SIRPA*, *Eqca-DQB4*
CH241-441N13	2	*Eqca-DQA1*, *-DQB1*	2	*Eqca-DQA4*, *-DQB4*
CH241-440J07	2	*Eqca-DQB1*, *-DQA2*	2	*Eqca-DQA4*, *-DRB4*
CH241-135M23	5	*Eqca-DQA2*, *-DQB2*, *-DQA3*, *-DQB3*, *-DRB2*	2	*RPS27*, *Eqca-DOB2*
CH241-455C07	2	*Eqca-DRB3, -DOB1*	3	*Eqca-DRB5*, *-DOB3*, *-DRB6,*
CH241-147K21	9	*Eqca-DOB1*, TAP2*, *PSMB8*, *TAP1*, *PSMB9*, *Eqca-DMB*, *-DMA*, *BRD2*, *Eqca-DOA**	0	-
CH241-359L18	8	*PSMB8*, *TAP1*, *PSMB9*, *Eqca-DMB*, *-DMA*, *BRD2*, *Eqca-DOA*, *COL11A2**	2	*Eqca-DPB1*, *-DPB2*

*partial gene

Table S4. Identity to known MHC-IPD alleles.

Gene	Most identical MHC-IPD allele	Accession No.	Nucleotide identity (%)
Eqca-DRA	*Eqca-DRA*00101*	JQ254080	100
Eqca-DRB1	*Eqca-DRB1*00101*	JQ254085	100
Eqca-DRB2	*Eqca-DRB2*00201*	JQ254091	99.6
Eqca-DRB3	*Eqca-DRB3*00201*	JQ254099	99.0
Eqca-DQA1	*Eqca-DQA1*00101*	JQ254060	100
Eqca-DQA2	*Eqca-DQA2*00101*	JQ254064	100
Eqca-DQA3	*Eqca-DQA3*00101*	JQ254068	100
Eqca-DQB1	*Eqca-DQB1*00101*	JQ254070	99.9
Eqca-DQB2	*Eqca-DQB2*00101*	JQ254075	100
Eqca-DQB3	*Eqca-DQB3*00101*	JQ287623	100

Supplementary Table S5. Primer sequences.

	**Forward primer 5'-3'**	**Reverse primer 5'-3'**
Amplicon 1	M13F-ccttgcatctggactcacct	M13R-ttgcccactgctgtattcac
Amplicon 2	M13F-ggtcaccagaggagaacagg	M13R-ttcctcagcagtggctttct
Candidate 1	M13F-gcaccaataacctgcaatgaa	M13R-ctctctcgttctctggattcca
Candidate 2	M13F-accagctgaccaccaatgta	M13R-caacaagaatgaggccctgg
Candidate 3	M13F-gcctattgccaaacattgcc	M13R-ttgtgtaagggagccaggag
Candidate 4	M13F-tgggacagcactattaatgtaca	M13R-agctttcactctcctcctct
Candidate 5	M13F-aggccaaggacacagatcag	M13R-tttcttctctctccctggcc
247 bp INDEL	gtttgctgcactccacttca	aggccagcttttgttttcatt

M13F: TGTAAAACGACGGCCAGT;
M13R:CAGGAAACAGCTATGACC.

Table S6. Detailed information of the sequences submitted to the European Nucleotide Archive.

ID	Run accession No	Sequencing technology
CH241-147K21	ERR1661471	PacBio
CH241-455C07	ERR1661476	PacBio
CH241-135M23	ERR1661475	PacBio
CH241-288J19	ERR1661473	PacBio
CH241-440J07	ERR1661470	PacBio
CH241-359L18	ERR1661472	PacBio
CH241-407K07	ERR1661469	PacBio
CH241-441N13	ERR1661474	PacBio
Het_Region_Nr1	ERR1661492	Sanger-sequencing
Het_Region_Nr2	ERR1661493	Sanger- sequencing
Het_Region_Nr3	ERR1661494	Sanger- sequencing
Het_Region_Nr4	ERR1661495	Sanger- sequencing
Het_Region_Nr5	ERR1661496	Sanger- sequencing
LR-PCR Amplicons	ERR1839600	PacBio
